# Chronic Lead Exposure and Tauopathies: Molecular Mechanisms of Neurotoxicity and Emerging Nanotherapeutic Strategies

**DOI:** 10.1155/jt/3248563

**Published:** 2026-10-13

**Authors:** Maryam Ebrahimi, Mahsa Javan, Mahdi Alijanianzadeh

**Affiliations:** ^1^ Faculty of Biological Sciences Kharazmi University Tehran Iran khu.ac.ir; ^2^ Faculty of Pharmacy Tabriz University of Medical Sciences Tabriz Iran tbzmed.ac.ir; ^3^ Department of Cell and Molecular Biology Faculty of Biological Sciences Kharazmi University Tehran Iran khu.ac.ir

**Keywords:** drug delivery, lead, nanotechnology, neurodegenerative diseases, neurotoxicity, tauopathies

## Abstract

Chronic exposure to lead (Pb) remains a critical global public health challenge, extending beyond its established neurotoxic effects to its emerging role in the pathogenesis of neurodegenerative disorders, particularly tauopathies. Accumulating evidence indicates that prolonged Pb exposure disrupts neuronal homeostasis through multiple interconnected mechanisms, including oxidative stress, mitochondrial dysfunction, neuroinflammation, epigenetic alterations, and dysregulation of tau phosphorylation. These molecular perturbations contribute to synaptic impairment, neuronal loss, and progressive cognitive decline. Current therapeutic strategies primarily rely on metal chelation and antioxidant interventions; however, their clinical efficacy is limited by poor blood–brain barrier penetration, nonselective metal removal, and systemic adverse effects. Recent advances in nanotechnology offer promising alternatives for targeted neuroprotection. Nanocarrier systems, including dendrimers, liposomes, polymeric nanoparticles, and micelles, enable enhanced brain delivery, controlled drug release, and reduced systemic toxicity. This review synthesizes current knowledge on the molecular mechanisms linking Pb exposure to tau‐related neurodegeneration and critically evaluates existing therapeutic approaches. Furthermore, it highlights the potential of nano‐based delivery systems to overcome the limitations of conventional therapies and outlines future directions for translational research in Pb‐associated neurodegenerative disorders.

## 1. Introduction

Lead (Pb) is a persistent environmental and occupational neurotoxicant released from industrial activities, contaminated products, and legacy paints and plumbing systems. Exposure occurs mainly through contaminated dust, water, soil, food, and occupational settings [1]. Children and developing fetuses are particularly vulnerable because of greater gastrointestinal absorption, immature nervous systems, frequent hand‐to‐mouth behavior, and placental transfer of Pb [2, 3]. Following absorption, Pb distributes to soft tissues and is subsequently stored in bone, which acts as a long‐term endogenous reservoir. Its gradual remobilization, particularly during periods of increased bone turnover, can sustain internal exposure and contribute to neurological, cardiovascular, renal, reproductive, hematological, and developmental toxicity [3, 4].

The central nervous system (CNS) is a major target of Pb toxicity. Developmental and chronic exposure has been associated with impaired learning and memory, hippocampal dysfunction, altered synaptic plasticity, glial activation, and neuronal injury [5, 6]. These effects are linked to disturbances in calcium‐dependent signaling, mitochondrial function, redox homeostasis, neurotransmission, and neuroimmune regulation [7]. Such alterations may also contribute to tau dysregulation, although their direct effects on specific tau‐regulating enzymes remain incompletely defined.

Tauopathies are neurodegenerative disorders characterized by abnormal modification, misfolding, aggregation, and intracellular accumulation of tau in neurons and, in some diseases, glial cells [8, 9]. They include primary tauopathies, such as progressive supranuclear palsy (PSP), corticobasal degeneration (CBD), Pick’s disease, and selected forms of frontotemporal dementia, as well as secondary tauopathies, most notably Alzheimer’s disease (AD), in which tau pathology occurs alongside amyloid‐β (Aβ) deposition [8, 9].

Experimental evidence indicates that Pb may promote pathological tau phosphorylation by disrupting the balance between tau‐regulating kinases and phosphatases, particularly glycogen synthase kinase‐3β (GSK‐3β), cyclin‐dependent kinase 5 (CDK5), and protein phosphatase 2A (PP2A) [10, 11].

Chelation therapy remains the conventional pharmacological approach for clinically significant Pb poisoning. Common agents include succimer, calcium disodium ethylenediaminetetraacetate, and dimercaprol, with treatment selected according to blood Pb concentration, clinical presentation, and poisoning severity [12]. However, lowering circulating Pb does not necessarily produce comparable brain clearance or reverse established neurological damage. Succimer reduces blood Pb concentrations, yet experimental and clinical studies have shown limited effects on brain Pb burden and neurodevelopmental outcomes [13, 14]. Conventional chelation is also constrained by adverse effects, limited selectivity, disruption of essential metal homeostasis, and the need for repeated administration [15].

These limitations have stimulated interest in biological nanochelation strategies designed to improve chelator pharmacokinetics, circulation time, and tissue targeting. In a chronic Pb‐poisoning model, red‐blood‐cell‐membrane–coated PLGA nanoparticles carrying dimercaptosuccinic acid (DMSA) increased systemic exposure and improved survival relative to free DMSA [16]. Nevertheless, the study did not establish effective blood–brain barrier (BBB) penetration, brain Pb clearance, or direct removal of intracellular neuronal Pb.

Functionalized liposomes, biodegradable polymeric and lipid nanoparticles, ligand‐modified nanocarriers, and engineered extracellular vesicles are increasingly being explored for CNS delivery through receptor‐mediated transcytosis, adsorptive transport, and biomimetic interactions [17–19]. These platforms could potentially enhance neuronal or glial delivery of chelators, support controlled intracellular release, and reduce nonspecific systemic exposure. However, direct evidence that BBB‐penetrating nanocarriers can selectively chelate intracellular or mitochondrial Pb in neurons remains scarce. Future studies should therefore demonstrate effective brain delivery, stable Pb complexation, preservation of essential metal homeostasis, safe elimination of Pb–chelator complexes, and improvement in tau‐related molecular and neurological outcomes.

Accordingly, this review examines the relationship between Pb exposure and tau‐associated neurodegeneration, with particular emphasis on Pb toxicokinetics, disruption of tau‐regulatory kinases and phosphatases, and the limitations of current chelation therapies. It further evaluates CNS‐targeted biological nanochelation strategies, including functionalized liposomes, extracellular vesicles, BBB‐crossing nanocarriers, and other biomimetic platforms. Environmental sensing and remediation approaches are considered only where their underlying design principles may inform biomedical Pb sequestration.

## 2. Tau Biology and Pb‐Induced Dysregulation of Tau‐Regulatory Pathways

Tau is an intrinsically disordered, microtubule‐associated protein encoded by MAPT. Alternative splicing generates six major isoforms in the adult human brain, classified as 3R or 4R tau according to the number of microtubule‐binding repeats. Although these isoforms are normally expressed at similar levels, individual tauopathies often show preferential accumulation of specific isoforms [20–22]. Their functional differences are relevant to Pb neurotoxicity, as most studies have assessed total or phosphorylated tau without distinguishing among isoforms.

Tau is regulated by several post‐translational modifications, particularly phosphorylation. Excessive or abnormal phosphorylation weakens tau–microtubule binding, promotes its mislocalization and aggregation, and disrupts axonal transport, mitochondrial function, synaptic activity, and neuronal survival. Tau pathology therefore reflects not only hyperphosphorylation but also altered localization, conformation, clearance, and intercellular spread [23–25].

Tau phosphorylation is controlled by the coordinated activity of several kinases and phosphatases. GSK‐3β and CDK5 phosphorylate tau at multiple disease‐relevant sites, whereas PP2A is responsible for a substantial proportion of tau dephosphorylation. Direct evidence that Pb disrupts this balance still comes mainly from earlier experimental studies. In rats exposed to Pb during the perinatal period, Pb increased tau phosphorylation at Ser396 and Ser199/202 and altered GSK‐3β activity and the calpain‐dependent conversion of p35 to p25, implicating both GSK‐3β– and CDK5‐related pathways [10]. Studies in developing rats and primary human fetal neurons also reported altered tau phosphorylation together with changes in the expression or activity of PP1, PP2A, PP2B, and PP5 [11, 26].

These studies remain mechanistically important, but their relevance to chronic human exposure is limited by differences in the developmental stage, exposure level, experimental duration, and the tau species examined. A recent review similarly concluded that the evidence for Pb‐induced disruption of tau‐directed kinases and phosphatases still relies heavily on these earlier experimental models, highlighting the need for confirmation in contemporary human‐relevant systems [27].

More recent work has broadened the proposed mechanism beyond kinase–phosphatase imbalance. In a 2024 study, adult C57BL/6J mice exposed to Pb in drinking water for ten months developed dose‐related impairments in learning and memory. These changes were accompanied by increased hippocampal tau mRNA, total tau, phosphorylated tau, and phosphorylated‐tau immunoreactivity. Pb exposure also reduced the expression of the circular RNA CDR1as, increased miR‐671 and AGO2 expression, and enhanced nuclear translocation of NF‐κB [28]. These findings suggest a possible link between chronic Pb exposure, noncoding RNA dysregulation, inflammatory signaling, and tau pathology. However, because CDR1as and miR‐671 were not experimentally manipulated, the study does not establish that this RNA pathway directly caused the observed tau changes.

A 2025 study provided further evidence that impaired tau clearance may contribute to Pb neurotoxicity. Using complementary cellular and animal models, Peng et al. showed that Pb increased reactive oxygen species and Keap1 expression, promoted Nrf2 degradation, and reduced downstream antioxidant proteins, including heme oxygenase‐1 and glutathione peroxidase. Pb also disrupted p62‐associated autophagic processing and promoted tau accumulation. Treatment with N‐acetylcysteine, artemisitene, or rapamycin attenuated several of these molecular and neurological changes [29]. These results suggest that Pb‐induced tau accumulation may reflect not only increased phosphorylation but also impaired proteostatic clearance caused by oxidative stress and defective autophagy.

Other recent studies have identified upstream processes that may create conditions favorable for tau dysregulation. Pb‐induced caspase‐1–dependent neuronal pyroptosis has been associated with neuroinflammation, disrupted calcium signaling, and impaired learning and memory in cellular and animal models [30]. Chronic Pb exposure has also been shown to disturb neuronal and astroglial energy metabolism, amino‐acid cycling, and mitochondrial substrate utilization in the mouse brain [31]. Separately, studies in experimental tauopathy models have demonstrated that microglial NF‐κB activation can promote tau spreading and toxicity [32]. Taken together, these findings provide a plausible connection between Pb‐induced inflammation, metabolic dysfunction, and tau pathology. Nevertheless, the Pb studies did not directly show that pyroptosis or metabolic disruption caused the formation, propagation, or toxicity of specific tau conformers.

### 2.1. Epigenetic and Noncoding RNA Regulation of Tau Following Pb Exposure

Recent evidence indicates that Pb exposure can alter the epigenetic landscape, although most human studies have examined peripheral tissues rather than the brain. In Pb‐exposed workers, miR‐148a expression was inversely associated with global DNA methylation, and DNMT1 was identified as a potential miR‐148a target [33]. Genomewide studies of occupationally exposed individuals have also identified Pb‐associated differentially methylated loci and increased DNA damage [34]. Changes in global or repetitive‐element methylation have additionally been reported in children, electronic waste workers, adults living near mining areas, and Pb‐recycling workers [35, 36]. Together, these studies show that Pb exposure is associated with altered DNA methylation. However, their cross‐sectional design and reliance on blood‐derived DNA limit conclusions about long‐term epigenetic changes in neurons or glial cells.

Animal studies provide stronger evidence that the epigenetic effects of developmental Pb exposure depend on tissue, sex, age, and exposure period. Perinatal exposure to environmentally relevant Pb concentrations produced sex‐ and tissue‐specific DNA methylation changes that remained detectable in adult mice, suggesting that blood methylation patterns may not accurately reflect epigenetic changes in other tissues [37]. Human birth‐cohort studies have associated prenatal Pb exposure with trimester‐ and sex‐specific differences in cord‐blood DNA methylation at birth [38, 39]. These findings suggest that the developing epigenome may be sensitive to prenatal Pb exposure; however, they do not establish the persistence of these alterations or demonstrate specific regulation of MAPT.

The clearest recent connection between Pb exposure, noncoding RNAs, and tau comes from the chronic adult‐mouse study described above. Pb exposure reduced CDR1as expression and increased miR‐671 and AGO2 expression, together with greater NF‐κB activation and tau phosphorylation [28]. Because CDR1as interacts with miRNAs and AGO2‐associated regulatory machinery, the authors proposed that disruption of the CDR1as/miR‐671 axis might contribute to the Alzheimer‐like molecular phenotype observed after Pb exposure. However, no CDR1as restoration, miR‐671 inhibition, AGO2‐binding analysis, or genetic rescue experiment was performed. The findings should therefore be interpreted as evidence of association rather than proof of direct post‐transcriptional regulation of MAPT.

Pb‐induced miRNA dysregulation may also affect other pathways involved in neurodegeneration. In adolescent mice, Pb exposure produced age‐dependent changes in miRNAs involved in β‐site APP‐cleaving enzyme 1 (BACE1) regulation and was accompanied by altered BACE1 expression [40]. This finding supports the broader view that Pb can disturb noncoding RNA networks related to Alzheimer‐type pathology. However, the study did not assess MAPT transcription, tau phosphorylation, or tau aggregation and should not be considered direct evidence of epigenetic tau regulation.

There is currently no direct evidence that Pb alters DNA methylation at the MAPT promoter, MAPT intron 0, or other regulatory regions of the gene. Recent studies in AD and related tauopathies have linked DNA methylation patterns to tau‐associated genes, brain tau burden, and cell‐type–specific transcriptional changes, but Pb exposure was not evaluated in these investigations [41, 42]. A 2026 integrative epigenomic study of 472 AD brains, for example, identified oligodendrocyte‐specific methylation and gene‐expression changes associated with the extent and biochemical state of tau pathology. These findings show that tau accumulation is accompanied by cell‐specific epigenomic alterations, but they do not identify Pb as a causal factor.

### 2.2. Potential Prion‐Like Propagation of Tau Following Pb Exposure

Pathological tau can adopt seed‐competent conformations and spread between cells, partly through extracellular vesicles (EVs). Recent studies have substantially strengthened the evidence for microglial involvement in this process. EVs isolated from human AD brain tissue transferred misfolded tau and induced endogenous tau pathology in cultured neurons and mouse brain [43]. In mouse models, plaque‐associated microglia increased EV release and accelerated tau propagation, while TREM2 deficiency enhanced the release, dispersion, and seeding activity of tau carried by microglial exosomes [44, 45].

Microglial NF‐κB signaling has also been shown to promote the release of seed‐competent tau and enhance tau spreading and toxicity in tauopathy models [32]. More recent structural analyses identified paired helical and straight tau filaments within EVs isolated from human AD brains and confirmed their seeding activity [46]. Consistent with these findings, a 2026 study using human iPSC–derived microglia showed that fibrillar tau resisted complete degradation and was subsequently released in seed‐competent forms, including within EVs capable of inducing neuronal tau aggregation [47].

However, none of these studies involved Pb exposure. Current Pb studies primarily report changes in MAPT expression, tau phosphorylation, kinase–phosphatase signaling, noncoding RNA regulation, and tau clearance. There is still no direct evidence that Pb generates seed‐competent tau species or promotes EV‐mediated tau propagation. This possibility therefore remains hypothetical and should be tested using tau‐biosensor assays, EV characterization, and Pb‐exposed human neuronal–microglial models.

### 2.3. Human Evidence and Knowledge Gaps

Human evidence linking Pb exposure to defined tauopathies remains extremely limited. No study identified to date has directly related measured Pb concentrations in blood, bone, cerebrospinal fluid, or brain tissue to neuropathologically confirmed PSP or CBD.

A case–control study in military veterans reported an association between firearm use and PSP, but firearm use was only an indirect surrogate for possible Pb exposure, and no Pb biomarkers were measured [48]. A 2021 review similarly identified firearm use and occupational exposure to metals as possible environmental risk factors for PSP, while emphasizing that the available evidence was inconsistent and insufficient to establish causality [49].

More recently, a large prospective analysis of 240 incident PSP diagnoses in the UK Biobank did not identify significant environmental exposure factors, although Pb exposure was not directly measured and the PSP diagnoses lacked neuropathological confirmation [50]. An earlier report of PSP‐like parkinsonism in a battery worker with long‐term occupational Pb exposure remains anecdotal and was not confirmed by neuropathological examination [51]. Comparable clinical or epidemiological evidence for CBD has not been reported.

Future studies should integrate validated measures of cumulative Pb exposure, including bone Pb, with plasma and cerebrospinal fluid phosphorylated tau, tau positron‐emission tomography, detailed phenotyping, and postmortem confirmation. Such studies are necessary to determine whether Pb contributes to specific human tauopathies or instead causes broader neurological injury without producing a defined tauopathy.

## 3. Lead Exposure

Lead (Pb) remains a major environmental and occupational toxicant. Current sources include deteriorating Pb‐based paint, contaminated dust, soil, water and food, mining, smelting, battery recycling, and electronic waste. Exposure occurs mainly through ingestion in the general population and inhalation in occupational settings [3]. After absorption, Pb is distributed to soft tissues and gradually accumulates in bone, which acts as a long‐term reservoir. Stored Pb may later re‐enter the circulation during pregnancy, lactation, aging, or other periods of increased bone turnover, prolonging internal exposure even after the external source has been removed [52].

Developing fetuses and children are particularly vulnerable because Pb crosses the placenta, is more readily absorbed during childhood, and can disrupt the developing nervous system. Exposure has been associated with reduced cognitive performance, poorer academic achievement, and attention‐related or behavioral problems [53–55]. In adults, cumulative Pb exposure may also contribute to cognitive decline and neurodegenerative processes, although evidence for a causal relationship with Parkinson’s disease remains limited [56].

### 3.1. Signs and Symptoms of Lead Poisoning

Neuropsychiatric symptoms can occur at blood Pb levels ranging from 25 to 60 μg/dL. These symptoms may include irritability, cognitive slowing, delayed reaction times, reduced concentration, slower motor nerve conduction, and headaches [57, 58]. Severe neuropsychiatric symptoms, including delirium, coma, seizures, severe headache, and encephalopathy (swelling of the brain and increased cranial pressure), can arise at blood Pb concentrations exceeding 100 μg/dL [59, 60]. Children may experience severe neuropsychiatric symptoms at lower thresholds than adults, typically at sustained blood Pb levels of 70 μg/dL or higher [61]. Chronic Pb exposure usually results in symptoms that develop over weeks to months, although brief but intense exposures can cause acute neurological and neuromuscular symptoms [62, 63]. Depending upon the duration and level of exposure, individuals with chronic Pb poisoning can experience a wide variety of symptoms, including nausea, abdominal pain, lack of coordination, depression, peripheral neuropathy (numbness and tingling in the extremities), impaired short‐term memory and concentration, fatigue, sleep disturbances, headache, stupor, slurred speech (confused mental state), and anemia [57, 64]. In children, chronic Pb poisoning may more commonly present with aggressive behavior and reluctance to play. The nervous system is particularly vulnerable to Pb toxicity in children, with more pronounced effects than in adults. This increased susceptibility is related to the greater sensitivity of children’s organs and tissues to Pb. In adults, chronic Pb exposure has been associated with diminished performance in some tests of cognitive and nervous system function [63, 65]. Even low levels of Pb exposure may worsen behavioral and learning problems, reduce IQ scores, and affect infants and young children, who are especially vulnerable to toxic effects [66–68].

### 3.2. Clinical Assessment and Biomarkers of Pb Exposure

Clinical assessment of Pb toxicity should combine exposure history, symptoms, and laboratory findings. Venous whole‐blood Pb remains the main biomarker for diagnosis and follow‐up, while urinary Pb may provide limited complementary information but should not replace blood measurement [69]. Because blood Pb mainly reflects recent exposure, it may underestimate the Pb stored in bone. Serial blood measurements and, where available, bone Pb analysis can better indicate cumulative exposure [70]. Renal, hepatic, and hematological tests may also help identify organ injury, whereas oxidative stress markers remain primarily research tools [71, 72]. No single biomarker fully reflects exposure duration, tissue burden, and clinical severity; therefore, results should be interpreted together.

## 4. Pb‐Induced Neurotoxicity

In animal studies, Pb exposure has been shown to produce a wide range of toxic effects on behavioral, physiological, and biochemical processes, damaging the central and peripheral nervous systems, cardiovascular system, hematopoietic system, kidneys, liver, and other organs [73]. The precise mechanisms underlying Pb neurotoxicity have not been fully established, but several key hallmarks are recognized, including disrupted neurotransmission, oxidative stress, interference with cellular signaling, and effects on membrane biophysics.

Pb also impairs energy metabolism by increasing protein kinase C activity in vascular cells and inhibiting the sodium–potassium pump. At the cellular level, Pb inhibits the normal release of calcium from mitochondria, promoting the formation of mitochondrial permeability transition pores and activating programmed cell death pathways [74]. In human cell models, Pb exposure impairs neuronal differentiation [75]. Animal experiments, including studies by Zhou et al. [76], have demonstrated that early life Pb exposure increases Aβ accumulation, amyloid plaque deposition, upregulation of amyloid precursor protein (APP), and disrupted cholesterol metabolism in the brain. Pb exposure also induces tau hyperphosphorylation and neuroinflammation in rodents, as well as Aβ accumulation in nonhuman primate models. Zebrafish studies have shown that Pb exposure alters gene expression and increases AD risk [77–79].

Structural impacts of Pb exposure include impaired axonal myelination, loss of gray matter in the prefrontal cortex, and early apoptotic neurodegeneration in adult rodents [75]. Chronic Pb exposure interferes with calcium (Ca^2+^) signaling cascades and calmodulin‐dependent kinase regulators, crucial for memory consolidation and synaptic plasticity [77, 80]. Perinatal Pb exposure disrupts numerous calmodulin‐dependent pathways, normally initiated by calcium and zinc [74]. In zebrafish, early developmental Pb exposure results in reduced axonal density, impaired cerebral transcriptomic pathway development, decreased neurogenesis and axonogenesis, and overall disruption of CNS ontogenesis [77, 80]. Some studies even suggest that such exposure may result in transgenerational changes in brain transcriptome expression.

Furthermore, Pb‐induced neurotoxicity involves BBB damage, aberrant synaptic proliferation, interference with thyroid hormone transport, and altered neurotransmitter release [75]. The dopaminergic (attention and executive function), glutamatergic (learning and memory) [75], and gamma‐aminobutyric acid (GABA)‐ergic systems are adversely affected, with Pb blocking N‐methyl‐D‐aspartate (NMDA) ion channels [74]. Due to its chemical similarity to calcium ions, Pb can cross the BBB and accumulate in regions such as the prefrontal cortex, hippocampus, and cerebellum, causing neurodegeneration, loss of nerve tissue, cognitive deficits, and behavioral changes. These changes are associated with the pathophysiology of conditions such as schizophrenia, Parkinson’s disease, and AD [74].

### 4.1. Mechanisms of Lead‐Induced Neurotoxicity

Pb is a well‐established neurotoxic element known to impair the CNS through multiple molecular and cellular mechanisms (Figure 1). Its toxic actions partly stem from its ability to mimic or replace essential metal ions, particularly divalent cations such as calcium (Ca^2+^), magnesium (Mg^2+^), and iron (Fe^2+^), as well as monovalent cations like sodium (Na^+^). By substituting for Ca^2+^, Pb can cross the BBB and enter neural tissue through voltage‐gated calcium channels (VGCCs), where it accumulates in astrocytes and neurons. This process contributes to neuronal injury in regions critical for cognition and coordination, such as the hippocampus, prefrontal cortex, and cerebellum. Furthermore, Pb‐induced disturbances in Na^+^ homeostasis interfere with sodium‐dependent physiological processes, including the generation of action potentials in excitatory neurons, synaptic neurotransmitter uptake (e.g., choline, dopamine, and gamma‐aminobutyric acid), and the regulation of Ca^2+^ uptake and storage within synaptosomes [57, 81].

**FIGURE 1:**
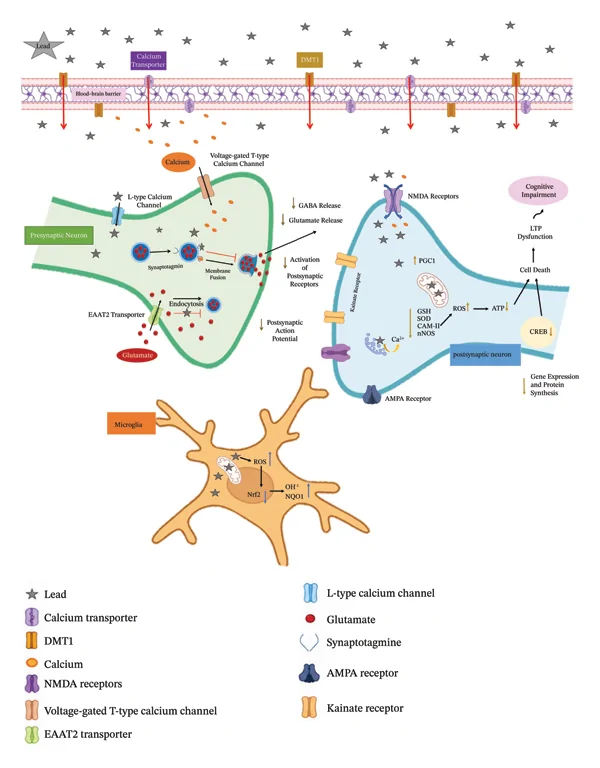
Lead (Pb) gains entry into the central nervous system primarily through divalent metal transporter 1 (DMT1) and calcium channel pathways. Within presynaptic neurons, Pb displays a stronger binding affinity for voltage‐gated calcium channels, thereby interfering with normal Ca^2+^ influx. Once inside the neuron, Pb interacts with Ca^2+^‐binding domains of proteins that regulate synaptic vesicle movement and docking, ultimately impairing neurotransmitter release and diminishing postsynaptic receptor activation. Moreover, Pb is capable of forming complexes with N‐methyl‐D‐aspartate (NMDA) receptors, altering intracellular Ca^2+^ homeostasis in postsynaptic neurons. Pb exposure also enhances the synthesis of kynurenic acid in astrocytes, a process that disrupts long‐term potentiation and synaptic plasticity. In parallel, Pb disturbs the redox equilibrium of neuronal cells, triggering oxidative stress and initiating cascades that culminate in neuronal apoptosis.

Pb also alters glutamatergic neurotransmission, the primary excitatory signaling pathway in the brain, by functioning as a noncompetitive and voltage‐dependent antagonist of NMDA receptors. These receptors act as nonselective cation channels that are essential for synaptic plasticity and the induction of long‐term potentiation (LTP), fundamental processes underlying learning and memory. Experimental findings in Pb‐exposed rodents have shown that synaptic responses mediated by both NMDA and α‐amino‐3‐hydroxy‐5‐methyl‐4‐isoxazolepropionic acid (AMPA) receptors are markedly disrupted, resulting in significant reductions in LTP. Such interference is accompanied by structural modifications in the dendritic architecture of CA1 pyramidal neurons within the hippocampus, thereby impairing cognitive functions and memory performance [81, 82].

Although Pb is redox‐inactive, it can indirectly induce oxidative stress by binding to sulfhydryl groups, depleting intracellular glutathione, and suppressing antioxidant enzymes, including superoxide dismutase, catalase, glutathione peroxidase, and glutathione reductase [57, 83]. Pb accumulation within mitochondria further disrupts calcium homeostasis, membrane potential, ATP synthesis, and electron transport chain function, thereby increasing ROS production and promoting neuronal injury [81, 84]. This mitochondrial–oxidative pathway provides an important mechanistic link between Pb accumulation, impaired cellular bioenergetics, and progressive neuronal dysfunction.

Both acute and chronic Pb exposures have been linked to the increased expression of genes associated with AD and their products, including APP and BACE1. BACE1, an aspartyl protease, cleaves APP, generating amyloidogenic peptides that can lead to Aβ plaque accumulation in brain tissue [85–87]. Continuous exposure to Pb has been linked to the suppression of low‐density lipoprotein receptor–related protein 1 (LRP1), a key member of the LDL receptor family located at the BBB. This receptor facilitates the efflux of Aβ peptides from the brain to the circulating blood. Its downregulation following Pb exposure reduces Aβ clearance efficiency, thereby promoting its accumulation within brain tissue [88].

Early life Pb exposure, encompassing both perinatal and neonatal periods, even at concentrations considered safe for humans, has been demonstrated to intensify tau‐related pathology. Pb enhances the expression and hyperphosphorylation of tau proteins at multiple serine and threonine residues by activating major tau kinases such as GSK‐3β and CDKs through calpain‐mediated proteolytic processes. These molecular events accelerate tau aggregation and neurofibrillary tangle (NFT) formation, disrupting cytoskeletal integrity and ultimately driving neuronal degeneration [10, 26].

Mitochondrial and oxidative injury induced by Pb can activate the NOD‐like receptor family pyrin domain‐containing 3 (NLRP3) inflammasome in microglia. Subsequent caspase‐1 activation promotes the release of interleukin‐1β and interleukin‐18, thereby amplifying neuroinflammatory signaling and contributing to learning and memory deficits [89, 90]. Pb also activates the TLR4–MyD88–NF‐κB pathway, promoting microgliosis, astrogliosis, and increased expression of inflammatory mediators, including TNF‐α, p38 MAPK, and ERK1/2 [91].

## 5. Current Management of Lead Toxicity

Current management of Pb toxicity focuses on reducing the body burden of the metal and limiting its systemic and neurological effects. Alongside established therapies, computational approaches such as virtual screening, molecular docking, molecular dynamics, and binding‐energy analysis may help identify candidate chelators, neuroprotective agents, and bioactive nanoplatforms, although experimental validation remains essential [87, 92–94]. The principal therapeutic approaches are outlined below.

### 5.1. Chelation Therapy

Chelation therapy is still the predominant treatment for heavy metal poisoning and remains the principal approach for managing Pb toxicity. Chelation therapy utilizes chelating agents, molecules that tightly bind to metal ions, forming stable complexes eliminated from the body, thereby reducing systemic toxicity. These agents function by coordinating the metal ion through one or more donor atoms or ligands, forming coordination compounds that can be classified as unidentate, bidentate, or polydentate based on their number of coordination sites. The selection of a chelating agent for clinical use primarily depends on the strength and stability of its binding with the metal ion, the ability to form nontoxic compounds, activity at physiological pH, and the capacity to penetrate the BBB in cases of neurotoxicity. Administration routes include intramuscular, intravenous, or oral delivery. While chelation therapy is effective and widely used, it does have limitations, such as nonselective binding, rapid clearance, poor membrane permeability, limited intracellular chelation, and potential adverse events like neurotoxicity or hypersensitivity reactions. Small molecule chelators often have short half‐lives and can be rapidly metabolized, sometimes allowing access to secondary infections [95]. Common chelating agents include dimercaprol (British antilewisite, BAL), 2,3‐dimercapto‐1‐propanesulfonic acid (DMPS), sodium–calcium–ethylenediaminetetraacetic acid (EDTA), deferoxamine (DFO), penicillamine, DMSA, and monoisoamyl DMSA (MiADMSA) [96].

In an experimental model, combined treatment with EDTA and *Ginkgo biloba* reduced Pb burden while attenuating associated oxidative and inflammatory injury [97]. EDTA promoted Pb complexation and elimination, whereas *G. biloba* provided complementary antioxidant and neuroprotective effects (Figure 2). However, these findings remain preclinical and do not establish this combination as a standard treatment for human Pb intoxication.

**FIGURE 2:**
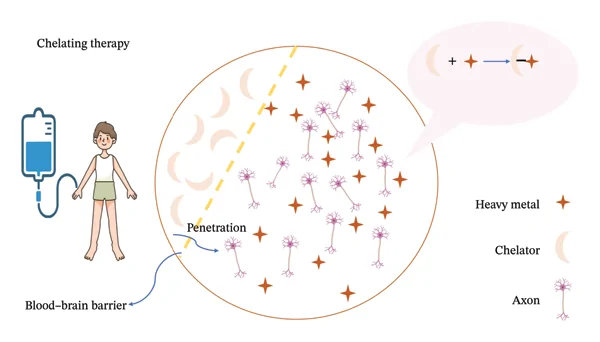
Schematic representation of chelation therapy for lead (Pb) toxicity, illustrating how chelating agents bind Pb ions to form stable complexes.

### 5.2. Antioxidant Therapy

Antioxidant therapy has been investigated as an adjunct to chelation for limiting secondary cellular and neuronal injury following Pb exposure. Compounds such as vitamins C and E, α‐lipoic acid, carnosine, and other antioxidant agents may reduce lipid peroxidation and support endogenous antioxidant defenses [98, 99]. Some agents may also provide additional anti‐inflammatory or neuroprotective effects. However, antioxidants do not directly remove Pb and should therefore be considered supportive rather than stand‐alone treatments. Clinical evidence regarding their optimal dose, treatment duration, and long‐term neurological benefits remains limited (Figure 3).

**FIGURE 3:**
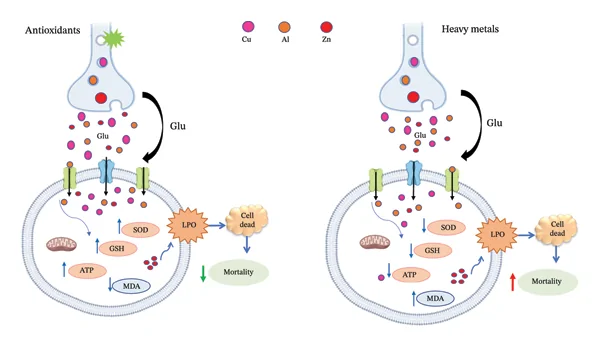
Illustration of antioxidant therapy in mitigating heavy metal‐induced oxidative stress. Antioxidants, including vitamins C and E, β‐carotene, alpha‐lipoic acid, as well as neuroprotective agents like carnosine and mexidol, scavenge free radicals and reduce metal deposition, thereby protecting cells from Pb‐induced damage.

### 5.3. Hemodialysis

Pb exposure can compromise renal function, particularly after acute high‐dose or prolonged exposure, and may contribute to tubular injury and disorders such as Fanconi syndrome. In severe cases, hemodialysis may be used in combination with chelation therapy to enhance the removal of circulating and chelatable Pb through complementary mechanisms (Figure 4) [100]. However, its effectiveness is limited because most Pb is distributed within tissues rather than remaining freely available in the bloodstream. Moreover, Pirsaheb et al. reported that reverse‐osmosis systems used in dialysis settings were ineffective in removing Pb and cadmium, highlighting the restricted role of dialysis‐based approaches in Pb detoxification [101].

**FIGURE 4:**
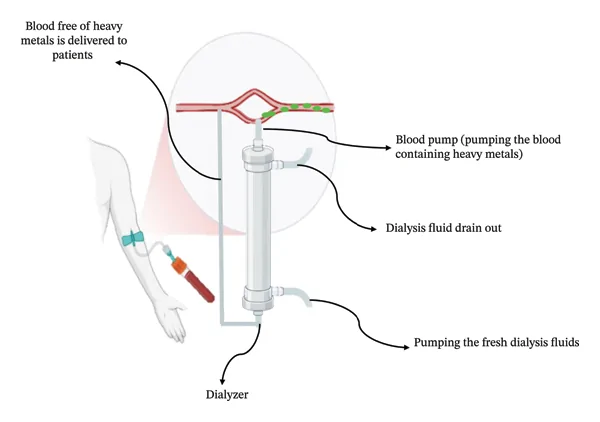
Schematic representation of hemodialysis in the removal of heavy metals. Hemodialysis facilitates the clearance of nephrotoxic metals, such as lead, from the bloodstream, helping to mitigate acute and chronic kidney injury associated with metal exposure.

### 5.4. Sauna Therapy

Sweating has been considered a potential complementary pathway for the elimination of toxic metals, including Pb. Detectable amounts of Pb have been reported in sweat, although concentrations vary according to exposure history, individual physiological factors, and the method of sample collection [102]. Water‐filtered infrared‐A sauna therapy has similarly been explored as a means of enhancing metal excretion through perspiration [103]. Nevertheless, the available clinical evidence is limited, and sauna therapy should be viewed only as an adjunctive approach rather than an established treatment for Pb intoxication.

## 6. Nanoenabled Chelation and CNS‐Targeted Delivery for Pb Neurotoxicity

Conventional chelation therapy can reduce circulating Pb levels but remains less effective against Pb retained within the CNS. Its therapeutic performance is limited by poor BBB penetration, rapid systemic clearance, nonselective binding to essential metal ions, and restricted access to intracellular Pb deposits. These limitations are particularly important in chronic Pb exposure, in which the metal may persist within neurons, glial cells, and subcellular compartments despite reductions in blood Pb concentrations [7, 12, 52]. Nanotechnology offers a potential strategy for overcoming these barriers by incorporating chelating agents into carriers that improve their pharmacokinetic stability, cellular uptake, and delivery to neural tissues. Liposomes, polymeric nanoparticles, dendrimers, and extracellular vesicles can be engineered to protect chelators from premature degradation, prolong systemic circulation, and promote transport across the BBB. Surface functionalization may further support brain‐specific uptake and reduce nonselective interactions with peripheral tissues [17, 18, 104]. Accordingly, the therapeutic relevance of nanotechnology in Pb neurotoxicity lies primarily in biological nanochelation and CNS‐targeted delivery rather than in the environmental adsorption or detection of Pb.

### 6.1. Systemic Nanochelation of Circulating Pb

Systemic nanochelation aims to reduce the circulating Pb pool and limit its continued redistribution to vulnerable organs. This approach is particularly relevant because a substantial proportion of blood Pb is associated with erythrocytes and hemoglobin, which restricts its accessibility to freely circulating chelators. Nanostructured chelating systems and magnetic adsorbents may improve contact with erythrocyte‐bound Pb, enhance metal‐binding capacity, and facilitate its controlled removal from the bloodstream [105, 106]. Fe_3_O_4_ nanoparticles coated with mesoporous silica shells have been developed for the removal of Pb from blood. These nanoparticles were designed to enter erythrocytes without inducing substantial hemolysis or coagulation and achieved approximately 73.5% Pb removal within 30 min. Functionalization with DMSA further improved their Pb‐binding capacity [106]. Similarly, DMSA‐modified Fe_3_O_4_ magnetic nanomotors exhibited an adsorption efficiency of 80.6% under an alternating magnetic field, compared with 40.6% under static conditions. In an extracorporeal circulation model in pigs, this system progressively reduced blood Pb concentrations to 0.098 ppm [105]. Magnetic mesoporous silica/ε‐polylysine nanomotors have also been designed to enhance contact with Pb‐contaminated hemoglobin through magnetically controlled movement within erythrocytes, while maintaining favorable cytocompatibility and blood compatibility [107]. In addition, BODIPY‐functionalized magnetic silica nanoparticles combine selective Pb recognition with magnetic separation, providing a potential platform for the simultaneous detection and removal of Pb from blood [108]. Despite these promising findings, current systemic nanochelation systems primarily target circulating or erythrocyte‐associated Pb. Evidence that these platforms can cross the BBB, enter neurons, or remove intracellular Pb from neural tissue remains limited. Therefore, systemic nanochelation should be considered a supportive strategy for reducing peripheral Pb burden, whereas dedicated brain‐targeted nanocarriers are required to address Pb‐associated neurodegeneration [16, 17]. Representative nanoparticle‐based approaches for Pb removal from contaminated samples are illustrated in Figure 5.

**FIGURE 5:**
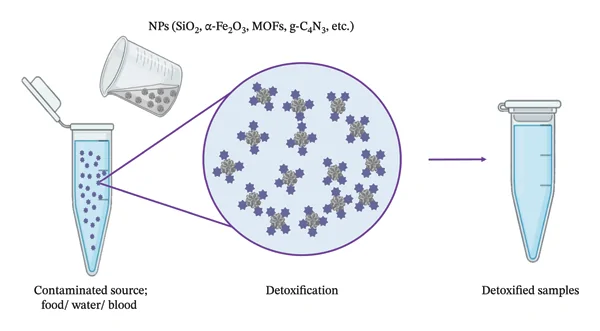
Schematic illustration of lead (Pb) removal from contaminated samples (food, water, or blood) using nanoparticles. Different nanoparticles (e.g., SiO_2_, α‐Fe_2_O_3_, MOFs, and g‐C_4_N_3_) were added to the contaminated medium, leading to the adsorption and detoxification of Pb^2+^ ions. After interaction with the nanoparticles, the detoxified samples were obtained with significantly reduced Pb content.

### 6.2. Biological Strategies for Pb Detoxification

Reducing Pb concentrations in blood does not necessarily result in the removal of Pb retained within the CNS. This distinction is particularly important in chronic exposure, in which Pb may persist in neural tissues despite an apparent reduction in circulating metal levels. Conventional chelators generally exhibit limited penetration across the BBB, rapid systemic clearance, and insufficient access to Pb deposited within the brain. Consequently, systemic chelation may reduce the peripheral Pb pool while leaving a substantial fraction of the neurotoxic burden untreated. Experience with nanoparticle‐assisted brain metal chelation provides a useful proof of principle that nanoscale carriers can improve the delivery of metal‐binding agents to the CNS; however, comparable evidence for Pb‐specific brain chelation remains limited [12, 16]. The BBB is formed by tightly connected brain endothelial cells supported by pericytes, astrocytic end‐feet, and extracellular matrix components. This structure restricts the passive entry of most hydrophilic chelators and macromolecular therapeutic agents. Although Pb exposure may impair BBB integrity, such disruption is heterogeneous and should not be regarded as a predictable route for therapeutic delivery. Effective brain‐directed nanochelation therefore requires carriers that remain stable in the circulation, avoid rapid clearance by the mononuclear phagocyte system, interact appropriately with brain endothelial cells, and release their cargo only after reaching the CNS. Nanoparticle size, morphology, surface charge, ligand density, and protein‐corona formation can all influence vascular residence, endothelial uptake, and subsequent transport into brain tissue [17, 18, 109]. Nanocarriers may cross the BBB through several transport pathways. Receptor‐mediated transcytosis is among the most widely investigated approaches and involves functionalizing the carrier surface with ligands that interact with receptors expressed on brain endothelial cells. Following receptor binding, the carrier is internalized, transported across the endothelial cell, and released on the brain‐facing side of the BBB. Adsorptive‐mediated transcytosis can also be promoted through controlled electrostatic interactions between positively charged carrier surfaces and the negatively charged endothelial membrane. However, strongly cationic nanoparticles may cause membrane injury, nonspecific protein adsorption, and systemic toxicity. Therefore, the surface properties of BBB‐directed nanocarriers must be optimized to balance endothelial interaction with biocompatibility and colloidal stability [17, 104]. Liposomes are particularly relevant to brain‐directed Pb chelation because their aqueous core can encapsulate hydrophilic chelators, whereas their phospholipid bilayer can accommodate lipophilic neuroprotective compounds. This structural versatility creates the possibility of combining Pb sequestration with the delivery of agents directed against Pb‐associated neuronal injury. Conventional liposomes, however, may undergo rapid clearance, cargo leakage, and limited BBB transport. PEGylation can prolong circulation by reducing opsonization and premature uptake by the liver and spleen, whereas surface functionalization with BBB‐targeting ligands may promote interaction with endothelial transport systems. Nevertheless, excessive PEG coverage may reduce cellular uptake and interfere with endosomal escape, while overly dense targeting ligands may increase nonspecific binding or alter nanoparticle stability. Therefore, the therapeutic performance of functionalized liposomes depends not only on the selected targeting ligand but also on lipid composition, particle size, membrane rigidity, surface density, and chelator‐release kinetics [110–112]. Poly(lactic‐co‐glycolic acid) nanoparticles represent another promising platform for brain‐directed chelator delivery. PLGA is a biodegradable polymer that can protect encapsulated compounds from premature degradation and provide sustained or stimulus‐responsive release. These characteristics may extend chelator exposure in brain tissue and reduce the need for repeated administration of rapidly cleared free agents. However, unmodified PLGA nanoparticles generally display limited BBB penetration. Their brain uptake is strongly influenced by size, surface charge, polymer composition, PEGylation, and the presence of targeting ligands. Surface‐engineered PLGA nanoparticles may therefore offer a means of improving endothelial transport while controlling the release of chelating or neuroprotective cargo after BBB passage. Potential limitations include initial burst release, incomplete drug loading, heterogeneous biodistribution, and the possibility that degradation kinetics may not match the time required for effective Pb complexation and clearance [18, 104].

Dendrimers offer a different design strategy based on a highly branched and multivalent architecture. Their internal cavities and numerous terminal functional groups allow chelators, imaging agents, targeting moieties, and neuroprotective compounds to be incorporated within a single nanosystem. This multifunctionality may be advantageous in Pb neurotoxicity, where the removal of the metal and attenuation of secondary neuronal injury may need to occur simultaneously. Chelating groups may be directly conjugated to the dendrimer surface or delivered as encapsulated cargo, while additional ligands can be introduced to enhance BBB interaction and neural‐cell uptake. However, dendrimer toxicity is strongly influenced by generation, molecular weight, surface charge, and terminal‐group chemistry. Highly cationic dendrimers may disrupt cellular membranes and induce hemolytic or neurotoxic effects; therefore, surface neutralization, partial acetylation, or PEGylation may be required before their use as CNS‐directed chelation platforms [113].

Polymeric micelles may also contribute to brain‐directed therapy, particularly when Pb chelation is combined with poorly soluble neuroprotective agents. Their hydrophobic core can improve the solubility and stability of lipophilic cargo, whereas the hydrophilic corona can prolong circulation and support surface functionalization. Nevertheless, conventional micelles are less suitable for highly hydrophilic chelators unless the chelating agent is chemically conjugated to the polymer or incorporated through ionic interactions. Dilution in the bloodstream may also destabilize micelles when their concentration falls below the critical micelle concentration. Thus, although micelles may be useful for adjunct neuroprotective delivery, liposomes, polymeric nanoparticles, and dendrimers currently provide more adaptable architectures for direct brain‐oriented nanochelation [114].

Importantly, successful BBB penetration should not be considered equivalent to effective Pb detoxification. A carrier may reach the brain but fail to release sufficient active chelator, or it may release the chelator extracellularly without reaching Pb retained inside neurons or glial cells. In addition, the resulting Pb–chelator complex must remain stable under physiological conditions and must ultimately be transported out of the brain or processed through a safe elimination pathway. Premature dissociation could redistribute Pb within neural tissue, whereas excessive chelator release could disturb the homeostasis of essential metals such as calcium, zinc, iron, and copper. An effective formulation must therefore combine selective Pb binding, controlled intracellular or extracellular release, minimal interference with essential metal ions, and a defined route for the elimination of the resulting metal complex [12, 115].

At present, most evidence supporting BBB‐penetrating nanocarriers is derived from general CNS drug‐delivery research or from nanoassisted chelation of metals other than Pb. Direct studies demonstrating that a Pb‐specific nanocarrier can cross the BBB, reduce Pb concentrations in defined brain regions, preserve essential metal homeostasis, and attenuate tau‐associated neurodegeneration remain scarce. Future investigations should therefore evaluate brain biodistribution, cellular localization, chelator‐release kinetics, Pb–chelator complex stability, neurological safety, and eventual clearance from neural tissue. Relevant therapeutic endpoints should extend beyond blood Pb reduction to include brain Pb burden, synaptic integrity, tau phosphorylation, neuronal viability, and cognitive function. Moreover, because Pb may remain within neurons and subcellular compartments after BBB passage, the next generation of nanocarriers must also be designed for effective cellular internalization and intracellular chelator release [16, 18, 115]. The structural and functional characteristics of these nanoplatforms are summarized in Table 1.

**TABLE 1:** Structural and functional characteristics of nanoplatforms with potential relevance to Pb detoxification and Pb‐induced CNS toxicity.

Nanoplatform	Current application	Key advantages	Key limitations and translational barriers	CNS/Pb evidence	Ref.
DMSA‐functionalized magnetic nanoparticles	Blood Pb removal and magnetic separation	Direct Pb binding; controllable separation	No BBB evidence; complete particle recovery is required	Pb removal shown in blood and extracorporeal models, but not in brain	[105, 106]
Functionalized liposomes	Therapeutic cargo delivery	Biocompatible; versatile cargo loading; clinically established platform	Limited stability and passive BBB transport; Pb‐specific efficacy is unproven	Potential for CNS delivery; no direct brain Pb‐removal study	[112, 116]
PLGA nanoparticles	Controlled therapeutic delivery	Biodegradable; sustained drug release	Limited intrinsic BBB transport; possible burst release	No direct evidence for brain Pb detoxification	[117, 118]
Dendrimers	Multivalent drug and chelator delivery	High loading and surface functionalization	Charge‐dependent cytotoxicity; complex synthesis	Potential CNS carrier; no direct Pb‐removal evidence	[119]
Exosomes/extracellular vesicles	Biomimetic CNS delivery	Natural carrier; can be engineered for brain targeting	Heterogeneity, low loading, and scale‐up challenges	Brain delivery shown experimentally; no Pb‐specific evidence	[120]
Chitosan nanoparticles	Drug delivery and metal adsorption	Biocompatible and mucoadhesive	Variable size and stability; BBB transport is uncertain	Pb evidence mainly relates to non‐CNS adsorption	[121–123]
Carbon nanotubes	Mainly sensing, adsorption, and experimental delivery	High surface area and cellular uptake	Persistence and inflammatory toxicity limit in vivo use	Mainly laboratory‐based; no validated in vivo Pb detoxification	[124, 125]
Quantum dots	Sensing and fluorescence imaging	High detection sensitivity and imaging capability	Composition‐dependent toxicity and uncertain clearance	No established therapeutic role in Pb chelation	[126, 127]

Magnetic nanoparticles currently provide the most direct evidence for biological Pb removal, although their use has mainly focused on circulating Pb. Liposomes, PLGA nanoparticles, and exosomes may be more suitable for CNS delivery, but their Pb‐specific efficacy remains unproven. In contrast, carbon nanotubes and quantum dots are primarily useful for sensing and imaging, while safety and clearance concerns limit their therapeutic translation.

### 6.3. Intracellular Targeting of Pb in Neurons

Crossing the BBB is necessary but not sufficient for effective brain Pb detoxification. After entering neural tissue, Pb can accumulate within neurons and glial cells, interact with sulfhydryl‐containing proteins, disrupt calcium‐dependent signaling, and localize within mitochondria. These intracellular pools are poorly accessible to conventional hydrophilic chelators and may persist even after circulating Pb concentrations decline [7, 31, 128]. Therefore, brain‐directed nanocarriers should support not only BBB transport but also cellular internalization and controlled intracellular chelator release.

Following entry into the brain, nanocarriers may be internalized by neurons through receptor‐mediated or adsorptive endocytosis. However, endocytosed carriers are commonly retained within endosomes and lysosomes, where premature degradation or sequestration can restrict access of the chelator to cytosolic and organellar Pb. Effective intracellular nanochelation therefore requires controlled cargo release and, where necessary, endosomal escape. Particle size, surface charge, ligand density, and carrier composition should be optimized to improve neuronal uptake without causing membrane damage or nonspecific accumulation [115, 129].

Mitochondrial Pb represents a particularly important therapeutic target because Pb‐associated mitochondrial dysfunction promotes calcium imbalance, membrane depolarization, impaired ATP production, and excessive reactive oxygen species generation [31, 128]. Nanocarriers designed to release chelators near mitochondria may improve access to this intracellular metal pool. Nevertheless, mitochondrial targeting must be carefully controlled because excessive carrier accumulation or highly cationic surface groups may themselves impair mitochondrial function.

Intracellular Pb removal may also be combined with neuroprotective therapy. Codelivery of a chelator with antioxidant or anti‐inflammatory agents could simultaneously reduce the metal burden and limit secondary injury caused by oxidative stress, microglial activation, and inflammatory signaling. This strategy may also be relevant to tau pathology because Pb exposure activates tau‐related kinases, including GSK‐3β and CDK5, and promotes tau hyperphosphorylation [28–30]. Thus, multifunctional nanocarriers could be designed to couple intracellular Pb sequestration with suppression of oxidative, inflammatory, and tau‐associated pathways.

Several safety requirements must be addressed. The intracellular chelator should preferentially bind Pb without markedly disrupting essential calcium, zinc, iron, or copper homeostasis. The resulting Pb–chelator complex must remain stable and should be transported out of the neuron or cleared from brain tissue. Otherwise, intracellular chelation could merely redistribute Pb between cellular compartments. Carrier degradation, neuronal toxicity, long‐term retention, and the possibility of Pb release from unstable complexes should therefore be evaluated [12, 115]. Direct evidence for the nanoparticle‐mediated removal of intracellular Pb from neurons remains scarce. Most available studies address circulating Pb removal or general CNS drug delivery rather than neuronal Pb extraction. Future studies should quantify intracellular and mitochondrial Pb, demonstrate stable Pb complex formation, evaluate essential metal preservation, and assess downstream outcomes such as mitochondrial function, tau phosphorylation, synaptic integrity, and neuronal survival [16, 18]. Despite this evidence gap, nanocarrier studies in AD and PD provide proof‐of‐concept evidence that engineered delivery systems can improve BBB transport, intracellular cargo delivery, and neuroprotective activity. However, these findings should not be interpreted as direct evidence for Pb detoxification. Representative nanocarrier‐based formulations investigated in AD models are summarized in Supporting Table S1 [130–154]. Corresponding nanocarrier‐based formulations investigated in PD models are summarized in Supporting Table S2 [155–167]. These studies may guide the development of Pb‐specific platforms that combine intracellular chelation with antioxidant, anti‐inflammatory, and antitau interventions.

## 7. Conclusion

Pb exposure remains an important global health concern because of its well‐established neurotoxicity and its possible contribution to neurodegenerative processes including tau‐related disorders. Current evidence suggests that Pb may disrupt tau homeostasis through interconnected pathways involving oxidative stress, mitochondrial dysfunction, neuroinflammation, epigenetic alterations, and dysregulation of GSK‐3β, CDK5, and tau‐directed phosphatases. However, direct evidence that Pb initiates tau seeding, promotes intercellular propagation, or contributes to specific primary tauopathies remains limited.

Conventional chelation mainly reduces the systemic Pb burden and is less effective against Pb retained within the CNS. Its therapeutic value may therefore be limited by poor BBB penetration, insufficient intracellular access, and a lack of selectivity for neural tissues. Brain‐targeted nanocarriers, including polymeric nanoparticles, functionalized liposomes, dendrimers, biomimetic systems, and extracellular vesicles, may offer a way to improve the delivery of chelators and complementary neuroprotective agents. Their clinical translation, however, will depend on a structured development pathway that addresses manufacturing, safety, biological performance, and patient selection.

From a regulatory perspective, candidate formulations must demonstrate reproducible large‐scale production, physicochemical stability, standardized characterization, and compliance with good manufacturing practice. Critical quality attributes, acceptable batch‐to‐batch variability, and validated potency assays should be defined early in development. Preclinical assessment should also extend beyond short‐term efficacy to include immunogenicity, hemocompatibility, pharmacokinetics, BBB transport, and interactions with neural and immune cells.

The long‐term fate of the nanocarrier itself is equally important. Future studies should examine its degradation, clearance, persistence, and potential accumulation in the brain, liver, kidneys, and spleen. Repeated‐dose studies will be needed to identify delayed neuroinflammation, organ toxicity, harmful degradation products, and adverse effects arising from the carrier rather than its therapeutic cargo. Demonstrating sustained therapeutic benefit without long‐term material accumulation will be essential before clinical testing.

Another major challenge is identifying which Pb‐exposed individuals are most likely to develop tau‐related cognitive decline. Prospective studies should evaluate biomarker panels combining blood and bone Pb burden with phosphorylated‐tau species, neurofilament light chain, glial and inflammatory markers, neuroimaging findings, and Pb‐associated epigenetic signatures. These panels should be validated longitudinally to distinguish cumulative exposure from active neuronal injury, identify high‐risk individuals, and monitor treatment response.

A practical translational roadmap should therefore proceed through three linked stages. The first is the standardization of nanocarrier production and demonstration of long‐term systemic and neurological safety. The second is confirmation of BBB penetration, neuronal uptake, intracellular Pb complexation, and safe removal of Pb–chelator complexes. The third is the clinical validation of biomarker‐guided strategies for selecting high‐risk patients and evaluating therapeutic response. Clear progression criteria should be established at each stage before moving toward human trials.

Future work should also determine whether Pb directly promotes tau seeding and intercellular propagation, clarify its effects on MAPT regulation, and quantify Pb burden in clinically and neuropathologically confirmed PSP, CBD, and other primary tauopathies. Addressing these priorities will be essential for translating CNS‐targeted nanotechnologies into safe, individualized, and clinically meaningful interventions for Pb‐associated neurodegeneration.

## Author Contributions

Maryam Ebrahimi: conceptualization and writing–original draft, review, and editing. Mahsa Javan: writing–review and editing, visualization, and resources. Mahdi Alijanianzadeh: supervision, review, and editing.

## Funding

No funding was received for this manuscript.

## Conflicts of Interest

The authors declare no conflicts of interest.

## Supporting Information

Additional supporting information can be found online in the Supporting Information section.

## Supporting information


**Supporting Information** Supporting Tables S1 and S2 summarize recent applications of nanoparticle‐based delivery systems for approved drugs investigated in AD and Parkinson’s disease models, respectively.

## Data Availability

The data that support the findings of this study are available from the corresponding author upon reasonable request.

## References

[1] Swaringen B. F. , Gawlik E. , Kamenov G. D. , McTigue N. E. , Cornwell D. A. , and Bonzongo J. C. J. , Children’s Exposure to Environmental Lead: A Review of Potential Sources, Blood Levels, and Methods Used to Reduce Exposure, Environmental Research. (2022) 204, 10.1016/j.envres.2021.112025. 34508773

[2] Sample J. , Lead Exposure in Children: Failure to Protect the Most Vulnerable, Journal of Pediatric Pharmacology and Therapeutics. (2024) 29, no. 3, 212–214, 10.5863/1551-6776-29.3.212. PMC11163901 38863859

[3] Parithathvi A. , Choudhari N. , and Dsouza H. S. , Prenatal and Early Life Lead Exposure Induced Neurotoxicity, Human & Experimental Toxicology. (2024) 43, 10.1177/09603271241285523. 39340316

[4] Samarghandian S. , Shirazi F. M. , Saeedi F. et al., A Systematic Review of Clinical and Laboratory Findings of Lead Poisoning: Lessons from Case Reports, Toxicology and Applied Pharmacology. (2021) 429, 10.1016/j.taap.2021.115681. 34416225

[5] Tobalu F. O. and Enogieru A. B. , Lead Neurotoxicity in Experimental Models: A Systematic Review on Effects on the Cerebrum, Cerebellum, and Hippocampus, Toxicology Reports. (2025) 14, 10.1016/j.toxrep.2025.102044. PMC12142343 40487381

[6] Carmona A. , Roudeau S. , and Ortega R. , Molecular Mechanisms of Environmental Metal Neurotoxicity: A Focus on the Interactions of Metals with Synapse Structure and Function, Toxics. (2021) 9, no. 9, 10.3390/toxics9090198. PMC8471991 34564349

[7] Virgolini M. B. and Aschner M. , Molecular Mechanisms of Lead Neurotoxicity, Advances in Neurotoxicology, 2021, Elsevier, 159–213. 10.1016/bs.ant.2020.11.002 PMC8276936 34263090

[8] Zhang X. , Wang J. , Zhang Z. , and Ye K. , Tau in Neurodegenerative Diseases: Molecular Mechanisms, Biomarkers, and Therapeutic Strategies, Translational Neurodegeneration. (2024) 13, no. 1, 10.1186/s40035-024-00429-6. PMC11302116 39107835

[9] Olfati N. , Shoeibi A. , and Litvan I. , Clinical Spectrum of Tauopathies, Frontiers in Neurology. (2022) 13, 10.3389/fneur.2022.944806. PMC9329580 35911892

[10] Gąssowska M. , Baranowska-Bosiacka I. , Moczydłowska J. et al., Perinatal Exposure to Lead (Pb) Promotes Tau Phosphorylation in the Rat Brain in a GSK-3β and CDK5 Dependent Manner: Relevance to Neurological Disorders, Toxicology. (2016) 347, 17–28, 10.1016/j.tox.2016.03.002. 27012722

[11] Rahman A. , Brew B. J. , and Guillemin G. J. , Lead Dysregulates serine/threonine Protein Phosphatases in Human Neurons, Neurochemical Research. (2011) 36, no. 2, 195–204, 10.1007/s11064-010-0300-6. PMC3023019 21046238

[12] Balali-Mood M. , Eizadi-Mood N. , Hassanian-Moghaddam H. et al., Recent Advances in the Clinical Management of Intoxication by Five Heavy Metals: Mercury, Lead, Chromium, Cadmium and Arsenic, Heliyon. (2025) 11, no. 4, 10.1016/j.heliyon.2025.e42696. PMC11876891 40040983

[13] Cremin Jr, J. , Luck M. , Laughlin N. , and Smith D. , Efficacy of Succimer Chelation for Reducing Brain Lead in a Primate Model of Human Lead Exposure, Toxicology and Applied Pharmacology. (1999) 161, no. 3, 283–293, 10.1006/taap.1999.8807. 10620486

[14] Rogan W. J. , Dietrich K. N. , Ware J. H. et al., The Effect of Chelation Therapy with Succimer on Neuropsychological Development in Children Exposed to Lead, New England Journal of Medicine. (2001) 344, no. 19, 1421–1426, 10.1056/nejm200105103441902. 11346806

[15] Andersen O. and Aaseth J. , A Review of Pitfalls and Progress in Chelation Treatment of Metal Poisonings, Journal of Trace Elements in Medicine and Biology. (2016) 38, 74–80, 10.1016/j.jtemb.2016.03.013. 27150911

[16] Wang H. , Yao Q. , Zhu W. et al., Biomimetic Antidote Nanoparticles: A Novel Strategy for Chronic Heavy Metal Poisoning, AAPS PharmSciTech. (2022) 24, no. 1, 10.1208/s12249-022-02466-8. 36451071

[17] Zha S. , Liu H. , Li H. , Li H. , Wong K. L. , and All A. H. , Functionalized Nanomaterials Capable of Crossing the blood–brain Barrier, ACS Nano. (2024) 18, no. 3, 1820–1845, 10.1021/acsnano.3c10674. PMC10811692 38193927

[18] Liu J. , Wang T. , Dong J. , and Lu Y. , The blood–brain Barriers: Novel Nanocarriers for Central Nervous System Diseases, Journal of Nanobiotechnology. (2025) 23, no. 1, 10.1186/s12951-025-03247-8. PMC11866817 40011926

[19] Bhom N. , Somandi K. , Ramburrun P. , and Choonara Y. E. , Extracellular Nanovesicles as Neurotherapeutics for Central Nervous System Disorders, Expert Opinion on Drug Delivery. (2025) 22, no. 1, 69–84, 10.1080/17425247.2024.2440099. 39644485

[20] Buchholz S. and Zempel H. , The Six Brain‐Specific TAU Isoforms and Their Role in Alzheimer’s disease and Related Neurodegenerative Dementia Syndromes, Alzheimer’s & Dementia. (2024) 20, no. 5, 3606–3628, 10.1002/alz.13784. PMC11095451 38556838

[21] Corsi A. , Bombieri C. , Valenti M. T. , and Romanelli M. G. , Tau Isoforms: Gaining Insight into MAPT Alternative Splicing, International Journal of Molecular Sciences. (2022) 23, no. 23, 10.3390/ijms232315383. PMC9735940 36499709

[22] Buchholz S. , Kabbani M. A. A. , Bell‐Simons M. et al., The Tau Isoform 1N4R Confers Vulnerability of MAPT Knockout Human Ipsc‐Derived Neurons to Amyloid Beta and Phosphorylated Tau‐Induced Neuronal Dysfunction, Alzheimer’s & Dementia. (2025) 21, no. 5, 10.1002/alz.14403. PMC12089071 40019008

[23] Hong X. , Huang L. , Lei F. et al., The Role and Pathogenesis of Tau Protein in Alzheimer’s Disease, Biomolecules. (2025) 15, no. 6, 10.3390/biom15060824. PMC12190531 40563464

[24] Nordbeck A. J. , Sharma B. , Garcia C. A. , and Tseng J. H. , Post-Translational Modifications as a Regulatory Code for Tau Function in Health and Disease, Frontiers in Dementia. (2026) 5, 10.3389/frdem.2026.1834737. PMC13272095 42318076

[25] Price T. M. , Tucker A. E. , and Funk K. E. , Molecular and Environmental Drivers of Tau Post-Translational Modifications and Tau Pathology, Ageing Research Reviews. (2026) 120, 10.1016/j.arr.2026.103223.42372976

[26] Rahman A. , Khan K. , Al-Khaledi G. , Khan I. , and Attur S. , Early Postnatal Lead Exposure Induces Tau Phosphorylation in the Brain of Young Rats, Acta Biologica Hungarica. (2012) 63, no. 4, 411–425, 10.1556/abiol.63.2012.4.1. 23134599

[27] Parithathvi A. , Harshitha P. , Mumbrekar K. D. , and Dsouza H. S. , Systematic Review on Neurotoxic Implications of Lead-Induced Gene Expression Alterations in the Etiology of Alzheimer’s Disease, Cellular and Molecular Neurobiology. (2025) 45, no. 1, 1–16, 10.1007/s10571-025-01613-6. PMC12595167 41201693

[28] Qiao M. , Yang H. , Liu L. et al., Chronic Lead Exposure in Adult Mice: Associations with miR-671/CDR1as Regulation, NF-κB Signaling, and Alzheimer’s Disease-Like Pathology, Toxics. (2024) 12, no. 6, 10.3390/toxics12060410. PMC11209093 38922090

[29] Peng D. , Wei P. , Li Z. , Wei R. , Li H. , and Li S. , The Role of P62/Nrf2/Keap1 Signaling Pathway in Lead‐Induced Neurological Dysfunction, CNS Neuroscience & Therapeutics. (2025) 31, no. 9, 10.1111/cns.70566. PMC12413483 40913351

[30] Peng D. , Wang L. , Fang Y. et al., Lead Exposure Induces Neurodysfunction Through caspase-1-Mediated Neuronal Pyroptosis, Environmental Research. (2024) 255, 10.1016/j.envres.2024.119210. 38795947

[31] Varadarajan K. , Bagga P. , Ramesh A. , Chugani A. N. , and Patel A. B. , Chronic Lead Exposure Disrupts Neurometabolic Activity in Mouse Brain: An Ex Vivo 1H-[13C]-NMR Study, Neurotoxicology. (2023) 94, 117–125, 10.1016/j.neuro.2022.11.005. 36368548

[32] Wang C. , Microglial NF-κB Drives Tau Spreading and Toxicity in a Mouse Model of Tauopathy, bioRxiv. (2021) . 10.1038/s41467-022-29552-6 PMC9005658 35413950

[33] Araujo M. L. d. , Gomes B. C. , Devóz P. P. et al., Association Between miR-148a and DNA Methylation Profile in Individuals Exposed to Lead (Pb), Frontiers in Genetics. (2021) 12, 10.3389/fgene.2021.620744. PMC7928366 33679885

[34] Meng Y. , Zhou M. , Wang T. et al., Occupational Lead Exposure on Genome-Wide DNA Methylation and DNA Damage, Environmental Pollution. (2022) 304, 10.1016/j.envpol.2022.119252. 35385786

[35] Yohannes Y. B. , Nakayama S. M. , Yabe J. et al., Methylation Profiles of Global LINE-1 DNA and the GSTP1 Promoter Region in Children Exposed to Lead (Pb), Epigenetics. (2022) 17, no. 13, 2377–2388, 10.1080/15592294.2022.2123924. PMC9665151 36131534

[36] Kalahasthi R. , Adepu V. K. , and Nagaraju R. , Global DNA Methylation and Its Association with Lead Exposure, B Vitamin Deficiency and Lifestyle Variables Among Lead-Recycling Workers in India, Public Health Toxicology. (2024) 4, no. 4, 1–9.

[37] Bailey A. , Nicholas B. , Darley R. et al., Characterization of the Class I MHC Peptidome Resulting from DNCB Exposure of Hacat Cells, Toxicological Sciences. (2021) 180, no. 1, 136–147, 10.1093/toxsci/kfaa184. PMC7916740 33372950

[38] Rygiel C. A. , Dolinoy D. C. , Perng W. et al., Trimester-Specific Associations of Prenatal Lead Exposure with Infant Cord Blood DNA Methylation at Birth, Epigenetics insights. (2020) 13, 10.1177/2516865720938669. PMC7372614 32734142

[39] Park J. , Kim J. , Kim E. , Kim W. J. , and Won S. , Prenatal Lead Exposure and Cord Blood DNA Methylation in the Korean Exposome Study, Environmental Research. (2021) 195, 10.1016/j.envres.2021.110767. 33515580

[40] Wang R. , Wu Z. , Liu R. et al., Age‐Related Mirnas Dysregulation and Abnormal BACE1 Expression Following Pb Exposure in Adolescent Mice, Environmental Toxicology. (2022) 37, no. 8, 1902–1913, 10.1002/tox.23536. 35426476

[41] Wei X. , Du P. , and Zhao Z. , Impacts of DNA Methylation on Tau Protein Related Genes in the Brains of Patients with Alzheimer’s Disease, Neuroscience Letters. (2021) 763, 10.1016/j.neulet.2021.136196. 34437990

[42] Oatman S. R. , Reddy J. S. , Atashgaran A. et al., Integrative Epigenomic Landscape of Alzheimer’s Disease Brains Reveals Oligodendrocyte Molecular Perturbations Associated with Tau, Nature Communications. (2026) 17, no. 1, 10.1038/s41467-026-68864-9. PMC12957399 41775686

[43] Ruan Z. , Pathak D. , Venkatesan Kalavai S. et al., Alzheimer’s Disease Brain-Derived Extracellular Vesicles Spread Tau Pathology in Interneurons, Brain. (2021) 144, no. 1, 288–309, 10.1093/brain/awaa376. PMC7880668 33246331

[44] Clayton K. , Delpech J. C. , Herron S. et al., Plaque Associated Microglia Hyper-Secrete Extracellular Vesicles and Accelerate Tau Propagation in a Humanized APP Mouse Model, Molecular Neurodegeneration. (2021) 16, no. 1, 10.1186/s13024-021-00440-9. PMC7986521 33752701

[45] Zhu B. , Liu Y. , Hwang S. et al., Trem2 Deletion Enhances Tau Dispersion and Pathology Through Microglia Exosomes, Molecular Neurodegeneration. (2022) 17, no. 1, 10.1186/s13024-022-00562-8. PMC9438095 36056435

[46] Fowler S. L. , Behr T. S. , Turkes E. et al., Tau Filaments are Tethered Within Brain Extracellular Vesicles in Alzheimer’s Disease, Nature Neuroscience. (2025) 28, no. 1, 40–48, 10.1038/s41593-024-01801-5. PMC11706778 39572740

[47] Karabova M. K. , del Ser‐Badia A. , Hedegaard A. et al., Tracking Tau and Cellular Responses in Human Ipsc-Microglia: From Uptake to Seedable Secretion, Including in Extracellular Vesicles, Alzheimer’s & Dementia. (2026) 22, no. 4, 10.1002/alz.71337. PMC13053939 41943483

[48] Kelley K. D. , Checkoway H. , Hall D. A. , Reich S. G. , Cunningham C. , and Litvan I. , Traumatic Brain Injury and Firearm Use and Risk of Progressive Supranuclear Palsy Among Veterans, Frontiers in Neurology. (2018) 9, 10.3389/fneur.2018.00474. PMC6020251 29973911

[49] Park H. K. , Ilango S. D. , and Litvan I. , Environmental Risk Factors for Progressive Supranuclear Palsy, Journal of Movement Disorders. (2021) 14, no. 2, 103–113, 10.14802/jmd.20173. PMC8175813 34062646

[50] Zhao C. W. , Dale M. , Pantelyat A. et al., Risk Factors for the Diagnosis of Progressive Supranuclear Palsy in the UK Biobank, Movement Disorders. (2026) 41, no. 7, 1727–1739, 10.1002/mds.70287. PMC13387903 42481001

[51] Sanz P. , Nogue S. , Vilchez D. , Vilchez J. , Casal A. , and Logroscino G. , Progressive Supranuclear Palsy-Like Parkinsonism Resulting from Occupational Exposure to Lead Sulphate Batteries, Journal of International Medical Research. (2007) 35, no. 1, 159–163, 10.1177/147323000703500119. 17408069

[52] Gonzalez-Villalva A. , Marcela R. L. , Nelly L. V. et al., Lead Systemic Toxicity: A Persistent Problem for Health, Toxicology. (2025) 515, 10.1016/j.tox.2025.154163. 40286900

[53] Heidari S. , Mostafaei S. , Razazian N. , Rajati M. , Saeedi A. , and Rajati F. , The Effect of Lead Exposure on IQ Test Scores in Children Under 12 years: A Systematic Review and Meta-Analysis of Case-Control Studies, Systematic Reviews. (2022) 11, no. 1, 10.1186/s13643-022-01963-y. PMC9150353 35637522

[54] Crawfurd L. , Todd R. , Hares S. , Sandefur J. , and Bonnifield R. S. , The Effect of Lead Exposure on Children’s Learning in the Developing World: A Meta-Analysis, The World Bank Research Observer. (2025) 40, no. 2, 229–260, 10.1093/wbro/lkae010.

[55] Rosenauer V. , Schwarz M. I. , Vlasak T. , and Barth A. , Childhood Lead Exposure Increases the Risk of Attention-Deficit-Hyperactivity Disorder: A Meta-Analysis, The Science of the Total Environment. (2024) 951, 10.1016/j.scitotenv.2024.175574. 39153625

[56] Zhao Y. , Ray A. , Portengen L. , Vermeulen R. , and Peters S. , Metal Exposure and Risk of Parkinson Disease: A Systematic Review and Meta-Analysis, American Journal of Epidemiology. (2023) 192, no. 7, 1207–1223, 10.1093/aje/kwad082. PMC10326611 37022311

[57] Flora G. , Gupta D. , and Tiwari A. , Toxicity of Lead: A Review with Recent Updates, Interdisciplinary Toxicology. (2012) 5, no. 2, 47–58, 10.2478/v10102-012-0009-2. PMC3485653 23118587

[58] Kosnett M. J. , Wedeen R. P. , Rothenberg S. J. et al., Recommendations for Medical Management of Adult Lead Exposure, Environmental Health Perspectives. (2007) 115, no. 3, 463–471, 10.1289/ehp.9784. PMC1849937 17431500

[59] O’Connor G. T. , Quinton H. B. , Kneeland T. et al., Median Household Income and Mortality Rate in Cystic Fibrosis, Pediatrics. (2003) 111, no. 4, e333–e339, 10.1542/peds.111.4.e333. 12671148

[60] Patrick L. , Lead Toxicity, a Review of the Literature. Part I: Exposure, Evaluation, and Treatment, Alternative Medicine Review. (2006) 11, no. 1. 16597190

[61] Ara A. and Usmani J. A. , Lead Toxicity: A Review, Interdisciplinary Toxicology. (2015) 8, no. 2. 10.1515/intox-2015-0009 PMC4961898 27486361

[62] Needleman H. , Lead Poisoning, Annual Review of Medicine. (2004) 55, no. 1, 209–222, 10.1146/annurev.med.55.091902.103653. 14746518

[63] Gidlow D. A. , Lead Toxicity, Occupational Medicine (London). (2015) 65, no. 5, 348–356, 10.1093/occmed/kqv170. 26187801

[64] Pearce J. , Burton’s Line in Lead Poisoning, European Neurology. (2007) 57, no. 2, 118–119, 10.1159/000098100. 17179719

[65] Lanphear B. P. , Hornung R. , Khoury J. et al., Erratum:“Low-Level Environmental Lead Exposure and Children’s Intellectual Function: An International Pooled Analysis, Environmental Health Perspectives. (2019) 127, no. 9, 10.1289/ehp5685. PMC6792371 31526192

[66] Canfield R. L. et al., Intellectual Impairment in Children with Blood Lead Concentrations Below 10 μg per Deciliter, New England Journal of Medicine. (2003) 348, no. 16, 1517–1526. 10.1056/NEJMoa022848 PMC4046839 12700371

[67] Sanders J. T. and Wyatt R. J. , IgA Nephropathy and Henoch–Schönlein purpura Nephritis, Current Opinion in Pediatrics. (2008) 20, no. 2, 163–170, 10.1097/mop.0b013e3282f4308b. 18332712

[68] Cowan L. , Esteban E. , McElroy-Hart R. et al., Binational Study of Pediatric Blood Lead Levels Along the United States/Mexico Border, International Journal of Hygiene and Environmental Health. (2006) 209, no. 3, 235–240, 10.1016/j.ijheh.2005.12.003. 16459142

[69] Olympio K. P. K. et al., Biomarkers of Lead Exposure: Platforms and Analysis, Biomarkers in Toxicology, 2023, Springer, 489–513.

[70] Zhang X. , Specht A. J. , Wells E. , Weisskopf M. G. , Weuve J. , and Nie L. H. , Evaluation of a Portable XRF Device for in Vivo Quantification of Lead in Bone Among a US Population, Science of the Total Environment. (2021) 753, 10.1016/j.scitotenv.2020.142351. PMC7677595 33207470

[71] Upadhyay K. , Viramgami A. , Bagepally B. S. , and Balachandar R. , Association Between Chronic Lead Exposure and Markers of Kidney Injury: A Systematic Review and Meta-Analysis, Toxicology Reports. (2024) 13, 10.1016/j.toxrep.2024.101837. PMC11664089 39717854

[72] Kolawole T. A. , Palacios J. , Husaini D. , and Nwokocha C. , Inflammation and Oxidative Stress Biomarkers in Heavy Metal Toxicity: Bridging the Gap to Personalized Clinical Interventions, Journal of Applied Toxicology. (2025) 45, no. 12, 2546–2579, 10.1002/jat.4874. 40813054

[73] Lee J.-W. , Choi H. , Hwang U. K. et al., Toxic Effects of Lead Exposure on Bioaccumulation, Oxidative Stress, Neurotoxicity, and Immune Responses in Fish: A Review, Environmental Toxicology and Pharmacology. (2019) 68, 101–108, 10.1016/j.etap.2019.03.010. 30884452

[74] Sharma P. , Chambial S. , and Shukla K. K. , Lead and Neurotoxicity, Indian Journal of Clinical Biochemistry. (2015) 30, no. 1, 1–2, 10.1007/s12291-015-0480-6. PMC4310833 25646035

[75] Meyer D. N. , Crofts E. J. , Akemann C. et al., Developmental Exposure to Pb2+ Induces Transgenerational Changes to Zebrafish Brain Transcriptome, Chemosphere. (2020) 244, 10.1016/j.chemosphere.2019.125527. PMC7015790 31816550

[76] Zhou C.-C. , Gao Z. Y. , Wang J. et al., Lead Exposure Induces Alzheimers’s Disease (AD)-Like Pathology and Disturbes Cholesterol Metabolism in the Young Rat Brain, Toxicology Letters. (2018) 296, 173–183, 10.1016/j.toxlet.2018.06.1065. 29908845

[77] Lee J. and Freeman J. L. , Embryonic Exposure to 10 μg L− 1 Lead Results in Female-Specific Expression Changes in Genes Associated with Nervous System Development and Function and Alzheimer’s disease in Aged Adult Zebrafish Brain, Metallomics. (2016) 8, no. 6, 589–596, 10.1039/c5mt00267b. 26776728

[78] Wu J. , Basha M. R. , Brock B. et al., Alzheimer’s disease (AD)-Like Pathology in Aged Monkeys After Infantile Exposure to Environmental Metal Lead (Pb): Evidence for a Developmental Origin and Environmental Link for AD, Journal of Neuroscience. (2008) 28, no. 1, 3–9, 10.1523/jneurosci.4405-07.2008. PMC2486412 18171917

[79] Lee J. and Freeman J. L. , Zebrafish as a Model for Investigating Developmental Lead (Pb) Neurotoxicity as a Risk Factor in Adult Neurodegenerative Disease: A Mini-Review, Neurotoxicology. (2014) 43, 57–64, 10.1016/j.neuro.2014.03.008. 24698670

[80] Zhang J. , Peterson S. M. , Weber G. J. , Zhu X. , Zheng W. , and Freeman J. L. , Decreased Axonal Density and Altered Expression Profiles of Axonal Guidance Genes Underlying Lead (Pb) Neurodevelopmental Toxicity at Early Embryonic Stages in the Zebrafish, Neurotoxicology and Teratology. (2011) 33, no. 6, 715–720, 10.1016/j.ntt.2011.07.010. PMC3225594 21839828

[81] Sanders T. , Liu Y. , Buchner V. , and Tchounwou P. , Neurotoxic Effects and Biomarkers of Lead Exposure: A Review, Reviews on Environmental Health. (2009) 24, no. 1, 15–46, 10.1515/reveh.2009.24.1.15. PMC2858639 19476290

[82] Wang T. , Guan R. L. , Liu M. C. et al., Lead Exposure Impairs Hippocampus Related Learning and Memory by Altering Synaptic Plasticity and Morphology During Juvenile Period, Molecular Neurobiology. (2016) 53, no. 6, 3740–3752, 10.1007/s12035-015-9312-1. 26141123

[83] Lopes A. C. B. A. et al., Lead Exposure and Oxidative Stress: A Systematic Review, Reviews of Environmental Contamination & Toxicology. (2016) 236, 193–238. 10.1007/978-3-319-20013-2_3 26423075

[84] Bandaru L. J. M. , Murumulla L. , and Challa S. , Exposure of Combination of Environmental Pollutant, Lead (Pb) and β-Amyloid Peptides Causes Mitochondrial Dysfunction and Oxidative Stress in Human Neuronal Cells, Journal of Bioenergetics and Biomembranes. (2023) 55, no. 1, 79–89, 10.1007/s10863-023-09956-9. 36637735

[85] Hampel H. , Vassar R. , De Strooper B. et al., The β-Secretase BACE1 in Alzheimer’s Disease, Biological Psychiatry. (2021) 89, no. 8, 745–756, 10.1016/j.biopsych.2020.02.001. PMC7533042 32223911

[86] Roselli S. , Satir T. M. , Camacho R. et al., APP-BACE1 Interaction and Intracellular Localization Regulate Aβ Production in iPSC-Derived Cortical Neurons, Cellular and Molecular Neurobiology. (2023) 43, no. 7, 3653–3668, 10.1007/s10571-023-01374-0. PMC10477112 37355492

[87] Zahed M. A. , Ebrahimi M. , Barmakhshad N. , Shemshadi S. , and Parsasharif N. , Mercury-Mediated Neurological Diseases: Insight into Molecular Mechanisms, Mutant Proteins, and Structure-Based Therapeutic Inhibitors, Toxicology and Environmental Health Sciences. (2024) 16, no. 4, 459–480, 10.1007/s13530-024-00238-4.

[88] Gu H. , Wei X. , Monnot A. D. et al., Lead Exposure Increases Levels of β-Amyloid in the Brain and CSF and Inhibits LRP1 Expression in APP Transgenic Mice, Neuroscience Letters. (2011) 490, no. 1, 16–20, 10.1016/j.neulet.2010.12.017. PMC3026879 21167913

[89] Su P. , Wang D. , Cao Z. , Chen J. , and Zhang J. , The Role of NLRP3 in Lead-Induced Neuroinflammation and Possible Underlying Mechanism, Environmental Pollution. (2021) 287, 10.1016/j.envpol.2021.117520. 34182382

[90] Zhu J. , Zhou F. , Zhou Q. et al., NLRP3 Activation in Microglia Contributes to Learning and Memory Impairment Induced by Chronic Lead Exposure in Mice, Toxicological Sciences. (2023) 191, no. 1, 179–191, 10.1093/toxsci/kfac115. 36308466

[91] Liu J.-T. , Chen B. Y. , Zhang J. Q. , Kuang F. , and Chen L. W. , Lead Exposure Induced Microgliosis and Astrogliosis in Hippocampus of Young Mice Potentially by Triggering TLR4–MyD88–NFκB Signaling Cascades, Toxicology Letters. (2015) 239, no. 2, 97–107, 10.1016/j.toxlet.2015.09.015. 26386401

[92] Ebrahimi M. , Karami L. , and Alijanianzadeh M. , Computational Repurposing Approach for Targeting the Critical Spike Mutations in B. 1.617. 2 (Delta), AY. 1 (Delta Plus) and C. 37 (Lambda) SARS-CoV-2 Variants Using Exhaustive Structure-Based Virtual Screening, Molecular Dynamic Simulations and MM-PBSA Methods, Computers in Biology and Medicine. (2022) 147, 10.1016/j.compbiomed.2022.105709. PMC9170597 35728285

[93] Ebrahimi M. and Alijanianzadeh M. , Evaluation of the Interaction Between Potent Small Molecules Against the Nipah Virus Glycoprotein in Malaysia and Bangladesh Strains, Accompanied by the Human Ephrin-B2 and Ephrin-B3 Receptors; a Simulation Approach, Molecular Diversity. (2024) 28, no. 2, 851–874, 10.1007/s11030-023-10624-8. PMC9939871 36808582

[94] Ebrahimi M. , Dodangeh F. , Javan M. , Doroudian M. , and Moradi M. , Curcumin-Based Nanomaterials for Dual-Target Inhibition of SARS-CoV-2 Omicron Main Protease and HMPV RNA Polymerase: A Computational Study, International Journal of Biological Macromolecules. (2026) 371, 10.1016/j.ijbiomac.2026.152984. 42269888

[95] Lv Q. , Lin J. , Huang H. et al., Nanosponge for Iron Chelation and Efflux: A Ferroptosis‐Inhibiting Approach for Myocardial Infarction Therapy, Advanced Science. (2024) 11, no. 25, 10.1002/advs.202305895. PMC11220697 38671590

[96] Kim J.-J. , Kim Y.-S. , and Kumar V. , Heavy Metal Toxicity: An Update of Chelating Therapeutic Strategies, Journal of Trace Elements in Medicine and Biology. (2019) 54, 226–231, 10.1016/j.jtemb.2019.05.003. 31109617

[97] Adebayo O. G. , Ben-Azu B. , Ajayi A. M. et al., Gingko Biloba Abrogate Lead-Induced Neurodegeneration in Mice Hippocampus: Involvement of NF-κB Expression, Myeloperoxidase Activity and Pro-Inflammatory Mediators, Biological Trace Element Research. (2022) 200, no. 4, 1–14, 10.1007/s12011-021-02790-3. 34240327

[98] Unsal V. , Dalkiran T. , Çiçek M. , and Kölükçü E. , The Role of Natural Antioxidants Against Reactive Oxygen Species Produced by Cadmium Toxicity: A Review, Advanced Pharmaceutical Bulletin. (2020) 10, no. 2, 184–202, 10.34172/apb.2020.023. PMC7191230 32373487

[99] Kulikova O. I. and Fedorova T. N. , Effects of Antioxidants on the Viability of the Human Neuroblastoma SH-SY5Y Cell Culture Under the Conditions of Heavy-Metal Toxicity, Biology and Medicine. (2016) 8, no. 4, 10.4172/0974-8369.1000305.

[100] Barbier O. , Jacquillet G. , Tauc M. , Cougnon M. , and Poujeol P. , Effect of Heavy Metals on, and Handling by, the Kidney, Nephron Physiology. (2005) 99, no. 4, p105–p110, 10.1159/000083981. 15722646

[101] Pirsaheb M. , Efficiency of Reverse Osmosis System in the Removal of Lead, Cadmium, Chromium and Zinc in Feed Water of Dialysis Instruments in Kermanshah Hospitals, Journal of Mazandaran University of Medical Sciences. (2014) 24, no. 118, 151–157.

[102] Sears M. E. , Kerr K. J. , and Bray R. I. , Arsenic, Cadmium, Lead, and Mercury in Sweat: A Systematic Review, Journal of Environmental and Public Health. (2012) 2012, no. 1, 184745–10, 10.1155/2012/184745. PMC3312275 22505948

[103] Cho K.-H. , Jung S. , Choi M. , Jung Y. , Lee C. G. , and Choi N. , Effect of Water Filtration Infrared-A (wIRA) Sauna on Inorganic Ions Excreted Through Sweat from the Human Body, Environmental Science and Pollution Research. (2023) 30, no. 7, 18260–18267, 10.1007/s11356-022-23437-3. PMC9546416 36207634

[104] Zhang W. , Mehta A. , Tong Z. , Esser L. , and Voelcker N. H. , Development of Polymeric Nanoparticles for blood–brain Barrier Transfer—Strategies and Challenges, Advanced Science. (2021) 8, no. 10, 10.1002/advs.202003937. PMC8132167 34026447

[105] Wang M. , Bao T. , Yan W. et al., Nanomotor-Based Adsorbent for Blood Lead (II) Removal in Vitro and in Pig Models, Bioactive Materials. (2021) 6, no. 4, 1140–1149, 10.1016/j.bioactmat.2020.09.032. PMC7588752 33134607

[106] Zhao Z. , Wang M. , Jiang X. , Lv R. , and Mao C. , Fe3O4 Nanoparticles Coated with Mesoporous Shells for Pb (II) Removal from Blood, ACS Applied Nano Materials. (2022) 5, no. 1, 249–258, 10.1021/acsanm.1c02739.

[107] Liu Z. , Xu T. , Wang M. , Mao C. , and Chi B. , Magnetic Mesoporous silica/ε-Polylysine Nanomotor-Based Removers of Blood Pb2+, Journal of Materials Chemistry B. (2020) 8, no. 48, 11055–11062, 10.1039/d0tb02270e. 33196725

[108] Koczorowski T. , Glowacka-Sobotta A. , Sysak S. et al., BODIPY-Based Nanomaterials—Sensing and Biomedical Applications, Applied Sciences. (2022) 12, no. 15, 10.3390/app12157815.

[109] Wu D. , Chen Q. , Chen X. , Han F. , Chen Z. , and Wang Y. , The blood–brain Barrier: Structure, Regulation and Drug Delivery, Signal Transduction and Targeted Therapy. (2023) 8, no. 1, 10.1038/s41392-023-01481-w. PMC10212980 37231000

[110] Jourdain M.-A. and Eyer J. , Recent Advances in Liposomes and Peptide-Based Therapeutics for Glioblastoma Treatment, Journal of Controlled Release. (2024) 376, 732–752, 10.1016/j.jconrel.2024.10.037. 39437968

[111] Du Q. , Liu Y. , Fan M. , Wei S. , Ismail M. , and Zheng M. , PEG Length Effect of peptide-Functional Liposome for Blood Brain Barrier (BBB) Penetration and Brain Targeting, Journal of Controlled Release. (2024) 372, 85–94, 10.1016/j.jconrel.2024.06.005. 38838784

[112] Yang Y. , Chu Y. , Li C. et al., Brain-Targeted Drug Delivery by in Vivo Functionalized Liposome with Stable D-Peptide Ligand, Journal of Controlled Release. (2024) 373, 240–251, 10.1016/j.jconrel.2024.07.014. 38977135

[113] Bharadwaj P. , Roullin V. G. , and Chain J. L. , Crossing the blood–brain Barrier: Advances in Dendrimer-Based Nanocarriers for Central Nervous System Delivery, Nanoscale. (2025) 17, no. 40, 23202–23227, 10.1039/d5nr02548f. 41031431

[114] Ghezzi M. , Pescina S. , Padula C. et al., Polymeric Micelles in Drug Delivery: An Insight of the Techniques for Their Characterization and Assessment in Biorelevant Conditions, Journal of Controlled Release. (2021) 332, 312–336, 10.1016/j.jconrel.2021.02.031. 33652113

[115] Liu J. , Cabral H. , and Mi P. , Nanocarriers Address Intracellular Barriers for Efficient Drug Delivery, Overcoming Drug Resistance, Subcellular Targeting and Controlled Release, Advanced Drug Delivery Reviews. (2024) 207, 10.1016/j.addr.2024.115239. 38437916

[116] Konig S. , Shen X. , Mantovani G. , Winkler G. S. , Zhu Z. , and Moeendarbary E. , Transferrin-Functionalized Liposomes Enhance MAPT-ASO Transport Across a 3D Blood–Brain Barrier Microvascular Network Model, International Journal of Molecular Sciences. (2025) 26, no. 23, 10.3390/ijms262311347. PMC12691827 41373505

[117] Joseph A. , Simo G. M. , Gao T. et al., Surfactants Influence Polymer Nanoparticle Fate Within the Brain, Biomaterials. (2021) 277, 10.1016/j.biomaterials.2021.121086. PMC8478896 34481289

[118] Madani F. , Morovvati H. , Webster T. J. et al., Combination Chemotherapy via Poloxamer 188 surface-Modified PLGA Nanoparticles that Traverse the blood–brain–barrier in a Glioblastoma Model, Scientific Reports. (2024) 14, no. 1, 10.1038/s41598-024-69888-1. PMC11341868 39174603

[119] Khiabani N. A. , Elmhirst A. R. J. , Dubey O. et al., Non-Invasive Brain Delivery of G4 70/30 PAMAM Dendrimers via the Intranasal Route, ACS Chemical Neuroscience. (2026) 17, no. 6, 1145–1158, 10.1021/acschemneuro.5c00787. PMC13003435 41804051

[120] Budhwani S. , Sreenivasamurthy S. G. S. , Garza K. H. et al., Exosome-Mediated Delivery of a PLD1 Modulator Overcomes Bioavailability Hurdles to Target Synaptopathy in Neurodegenerative Diseases, Pharmaceutical Research. (2025) 42, no. 10, 1805–1819, 10.1007/s11095-025-03944-x. PMC12768594 41087673

[121] Gabold B. , Adams F. , Brameyer S. et al., Transferrin-Modified Chitosan Nanoparticles for Targeted Nose-to-Brain Delivery of Proteins, Drug Delivery and Translational Research. (2023) 13, no. 3, 822–838, 10.1007/s13346-022-01245-z. PMC9892103 36207657

[122] Mujtaba M. A. , Rashid M. A. , Godbole M. D. et al., Escitalopram Oxalate-Loaded Chitosan Nanoparticle in Situ Gel Formulation Intended for Direct Nose-to-Brain Delivery: In Vitro, Ex Vivo, and in Vivo Pharmacokinetic Evaluation, Frontiers in Pharmacology. (2025) 16, 10.3389/fphar.2025.1577331. PMC11985459 40223921

[123] Algamdi M. , Alshahrani A. , and Alsuhybani M. , Chitosan Grafted Tetracarboxylic Functionalized Magnetic Nanoparticles for Removal of Pb (II) from an Aqueous Environment, International Journal of Biological Macromolecules. (2023) 225, 1517–1528, 10.1016/j.ijbiomac.2022.11.208. 36427619

[124] Soligo M. , Felsani F. M. , Da Ros T. et al., Distribution in the Brain and Possible Neuroprotective Effects of Intranasally Delivered multi-Walled Carbon Nanotubes, Nanoscale Advances. (2021) 3, no. 2, 418–431, 10.1039/d0na00869a. PMC9418508 36131737

[125] Mahmood U. , Alkorbi A. S. , Hussain T. et al., Adsorption of Lead Ions from Wastewater Using Electrospun zeolite/MWCNT Nanofibers: Kinetics, Thermodynamics and Modeling Study, RSC Advances. (2024) 14, no. 9, 5959–5974, 10.1039/d3ra07720a. PMC10867556 38362070

[126] Sharma V. and Mehata M. S. , Synthesis of Photoactivated Highly Fluorescent Mn2+-Doped ZnSe Quantum Dots as Effective Lead Sensor in Drinking Water, Materials Research Bulletin. (2021) 134, 10.1016/j.materresbull.2020.111121.

[127] Chen Y. , Sekioka K. , Mosleh N. et al., Dual-Emissive Carbon Dots for the Sensitive and Selective Detection of Lead Ions and Differentiation of Multiple Metal Ions, ACS Applied Optical Materials. (2025) 3, no. 12, 2935–2945, 10.1021/acsaom.5c00498.

[128] Wang Y. , Shen X. , Guan R. et al., Lead Induces Mitochondrial Dysregulation in SH-SY5Y Neuroblastoma Cells via a lncRNA/circRNA–miRNA–mRNA Interdependent Networks, International Journal of Molecular Sciences. (2025) 26, no. 14, 10.3390/ijms26146851. PMC12295882 40725099

[129] Desai N. , Rana D. , Salave S. , Benival D. , Khunt D. , and Prajapati B. G. , Achieving endo/lysosomal Escape Using Smart Nanosystems for Efficient Cellular Delivery, Molecules. (2024) 29, no. 13, 10.3390/molecules29133131. PMC11243486 38999083

[130] Mufamadi M. S. , Kumar P. , du Toit L. C. et al., Liposome-Embedded, Polymeric Scaffold for Extended Delivery of Galantamine, Journal of Drug Delivery Science and Technology. (2019) 50, 255–265, 10.1016/j.jddst.2019.02.001.

[131] Elhabak M. , Salama A. A. , and Salama A. H. , Nose-To-Brain Delivery of Galantamine Loaded Nanospray Dried Polyacrylic acid/taurodeoxycholate Mixed Matrix as a Protective Therapy in lipopolysaccharide-Induced Alzheimer’s in Mice Model, International Journal of Pharmaceutics. (2023) 632, 10.1016/j.ijpharm.2023.122588. 36623740

[132] Lotfi M. , Nakhlband A. , Doustmihan A. , Omidi Y. , Fathi M. , and Adibkia K. , Formulation and Evaluation of in-situ Forming Poloxamer-based Hydrogel Containing Thiolated Chitosan Nanoparticles as Intranasal Galantamine Delivery System, International Journal of Biological Macromolecules. (2025) 320, 10.1016/j.ijbiomac.2025.145647. 40588203

[133] Li W. , Zhou Y. , Zhao N. , Hao B. , Wang X. , and Kong P. , Pharmacokinetic Behavior and Efficiency of Acetylcholinesterase Inhibition in Rat Brain After Intranasal Administration of Galanthamine Hydrobromide Loaded Flexible Liposomes, Environmental Toxicology and Pharmacology. (2012) 34, no. 2, 272–279, 10.1016/j.etap.2012.04.012. 22613079

[134] Fornaguera C. , Feiner-Gracia N. , Calderó G. , García-Celma M. J. , and Solans C. , Galantamine-Loaded PLGA Nanoparticles, from nano-emulsion Templating, as Novel Advanced Drug Delivery Systems to Treat Neurodegenerative Diseases, Nanoscale. (2015) 7, no. 28, 12076–12084, 10.1039/c5nr03474d. 26118655

[135] Misra S. , Chopra K. , Sinha V. R. , and Medhi B. , Galantamine-Loaded solid–lipid Nanoparticles for Enhanced Brain Delivery: Preparation, Characterization, in Vitro and in Vivo Evaluations, Drug Delivery. (2016) 23, no. 4, 1434–1443, 10.3109/10717544.2015.1089956. 26405825

[136] Singh S. K. and Mishra D. N. , Nose to Brain Delivery of Galantamine Loaded Nanoparticles: In-Vivo Pharmacodynamic and Biochemical Study in Mice, Current Drug Delivery. (2019) 16, no. 1, 51–58. 10.2174/1567201815666181004094707 30289074

[137] Cesur S. , Cam M. E. , Sayin F. S. , and Gunduz O. , Electrically Controlled Drug Release of Donepezil and BiFeO3 Magnetic nanoparticle-loaded PVA microbubbles/nanoparticles for the Treatment of Alzheimer’s disease, Journal of Drug Delivery Science and Technology. (2022) 67, 10.1016/j.jddst.2021.102977.

[138] Tripathi S. , Donepezil Loaded PLGA Nanoparticles, from Modified nano-precipitation, an Advanced Drug Delivery System to Treat Alzheimer Disease. in Journal of Physics: Conference Series, 2021, IOP Publishing.

[139] Kömür M. , Kıyan H. T. , and Öztürk A. A. , Development of Donepezil hydrochloride-loaded PLGA-based Nanoparticles for Alzheimer’s Disease Treatment, Scientific Reports. (2025) 15, no. 1, 10.1038/s41598-025-95792-3. PMC12003670 40240764

[140] Garg Y. , Kumar M. , Sharma G. , Katare O. P. , Chopra S. , and Bhatia A. , Systematic Designing and Optimization of Polymeric Nanoparticles Using Central Composite Design: A Novel Approach for nose-to-brain Delivery of Donepezil Hydrochloride, Journal of Cluster Science. (2024) 35, no. 4, 1007–1019, 10.1007/s10876-023-02528-2.

[141] Parviz G. , Guler E. , Hazar-Yavuz A. N. et al., Characterization and Anti-alzheimer’s Effect of donepezil-loaded chitosan/polyethylene Glycol Nanospheres, Journal of Drug Delivery Science and Technology. (2025) 108, 10.1016/j.jddst.2025.106887.

[142] Parviz G. and Çam M. , Donepezil/Curcumin-Loaded Nanoparticles-Embedded Microneedles for the Treatment of Alzheimer’s Disease: In Vitro Evaluation, 2024.

[143] Kaboli Z. , Hosseini M. J. , Sadighian S. , Rostamizadeh K. , Hamidi M. , and Manjili H. K. , Valine Conjugated Polymeric Nanocarriers for Targeted co-delivery of Rivastigmine and Quercetin in Rat Model of Alzheimer Disease, International Journal of Pharmaceutics. (2023) 645, 10.1016/j.ijpharm.2023.123418. 37716484

[144] Rompicherla S. K. L. , Arumugam K. , Bojja S. L. , Kumar N. , and Rao C. M. , Pharmacokinetic and Pharmacodynamic Evaluation of Nasal Liposome and Nanoparticle Based Rivastigmine Formulations in Acute and Chronic Models of Alzheimer’s Disease, Naunyn-schmiedeberg’s Archives of Pharmacology. (2021) 394, no. 8, 1737–1755, 10.1007/s00210-021-02096-0. PMC8298375 34086100

[145] Wavikar P. , Pai R. , and Vavia P. , Nose to Brain Delivery of Rivastigmine by in Situ Gelling Cationic Nanostructured Lipid Carriers: Enhanced Brain Distribution and Pharmacodynamics, Journal of Pharmaceutical Sciences. (2017) 106, no. 12, 3613–3622, 10.1016/j.xphs.2017.08.024. 28923321

[146] El-Assal M. and Samuel D. , Optimization of Rivastigmine Chitosan Nanoparticles for Neurodegenerative Alzheimer; in Vitro and Ex Vivo Characterizations, International Journal of Pharmacy and Pharmaceutical Sciences. (2022) 14, no. 1, 17–27, 10.22159/ijpps.2022v14i1.43145.

[147] Basharzad S. F. , Hamidi M. , Maleki A. , Karami Z. , Mohamadpour H. , and Reza Saghatchi Zanjani M. , Polysorbate-Coated Mesoporous Silica Nanoparticles as an Efficient Carrier for Improved Rivastigmine Brain Delivery, Brain Research. (2022) 1781, 10.1016/j.brainres.2022.147786. 35041841

[148] Mousavi S. V. and Hashemianzadeh S. M. , Poly (n-butyl Cyanoacrylate) as a Nanocarrier for Rivastigmine Transport Across the blood–brain Barrier in Alzheimer’s Disease Treatment: A Perspective from Molecular Dynamics Simulations, Journal of Molecular Modeling. (2018) 24, no. 9, 10.1007/s00894-018-3799-0. 30145721

[149] Cunha S. , Swedrowska M. , Bellahnid Y. et al., Thermosensitive in Situ Hydrogels of rivastigmine-loaded Lipid-based Nanosystems for nose-to-brain Delivery: Characterisation, Biocompatibility, and Drug Deposition Studies, International Journal of Pharmaceutics. (2022) 620, 10.1016/j.ijpharm.2022.121720. 35413397

[150] Gao Y. , Almalki W. H. , Afzal O. et al., Systematic Development of Lectin Conjugated Microspheres for nose-to-brain Delivery of Rivastigmine for the Treatment of Alzheimer’s Disease, Biomedicine & Pharmacotherapy. (2021) 141, 10.1016/j.biopha.2021.111829. 34147904

[151] Saleh M. A. , Mohamed J. , Ruby J. et al., Preparation of memantine-loaded Chitosan Nanocrystals: In Vitro and Ex Vivo Toxicity Analysis, Crystals. (2022) 13, no. 1, 10.3390/cryst13010021.

[152] Rani V. , Verma R. , Kumar K. , and Chawla R. , pH-influenced self-assembled Stealth Nanoscaffolds Encapsulating Memantine for Treatment of Alzheimer’s Disease: Varsha Rani et al, Journal of Biosciences. (2023) 48, no. 3, 10.1007/s12038-023-00343-5. 37593987

[153] Rani V. and Chawla R. , Design, Fabrication, Optimization and Characterization of memantine-loaded Biodegradable PLGA Nanoscaffolds for Treatment of Alzheimer’s Disease, Biomedical Materials. (2022) 17, no. 6, 10.1088/1748-605x/ac9811. 36202081

[154] Kaur A. , Nigam K. , Tyagi A. , and Dang S. , A Preliminary Pharmacodynamic Study for the Management of Alzheimer’s Disease Using memantine-loaded PLGA Nanoparticles, AAPS PharmSciTech. (2022) 23, no. 8, 10.1208/s12249-022-02449-9. 36380129

[155] Liang K. , Yang L. , Kang J. et al., Improving Treatment for Parkinson’s disease: Harnessing Photothermal and phagocytosis-driven Delivery of Levodopa Nanocarriers Across the blood-brain Barrier, Asian Journal of Pharmaceutical Sciences. (2024) 19, no. 6, 10.1016/j.ajps.2024.100963. PMC11616058 39640059

[156] Kalčec N. , Peranić N. , Barbir R. et al., Spectroscopic Study of L-DOPA and Dopamine Binding on Novel Gold Nanoparticles Towards More Efficient drug-delivery System for Parkinson’s Disease, Spectrochimica Acta Part A: Molecular and Biomolecular Spectroscopy. (2022) 268, 10.1016/j.saa.2021.120707. 34902692

[157] Kalčec N. , Design and Characterization of Gold and Selenium Nanoparticles as Potential Delivery Systems of Levodopa and Dopamine, Sveučilište u Zagrebu, Sveučilište u Zagrebu Prirodoslovno-matematički. (2023) .

[158] Patel R. and Jain A. P. , Preparation and Characterization of Levodopa-Selegiline Loaded Chitosan Nanoparticles, International Journal of Medical Pharmaceutical and Health Sciences. (2024) 1, no. 1, 34–40, 10.62946/ijmphs/1.1.34-40.

[159] Guo X. , Qiao X. , Li X. et al., Lactoferrin-Modified organic-inorganic Hybrid Mesoporous Silica for co-delivery of Levodopa and Curcumin in the Synergistic Treatment of Parkinson’s disease, Phytomedicine. (2025) 140, 10.1016/j.phymed.2025.156547. 40054175

[160] Rukmangathen R. , Yallamalli I. M. , and Yalavarthi P. R. , Biopharmaceutical Potential of Selegiline Loaded Chitosan Nanoparticles in the Management of Parkinson’s disease, Current Drug Discovery Technologies. (2019) 16, no. 4, 417–425, 10.2174/1570163815666180418144019. 29669501

[161] Kumar S. , Dang S. , Nigam K. , Ali J. , and Baboota S. , Selegiline Nanoformulation in Attenuation of Oxidative Stress and Upregulation of Dopamine in the Brain for the Treatment of Parkinson’s disease, Rejuvenation Research. (2018) 21, no. 5, 464–476, 10.1089/rej.2017.2035. 29717617

[162] Katamesh A. A. , Abdel-Bar H. M. , Break M. K. B. et al., Tailored Intranasal Albumin Caged selegiline-α Synuclein siRNA Liposome with Improved Efficiency in Parkinson’s Model, Pharmaceutics. (2025) 17, no. 2, 10.3390/pharmaceutics17020243. PMC11859980 40006609

[163] Patil A. , Rajput A. , Subbappa P. , and Pawar A. , Formulation, Development and in Vivo Characterization of Selegiline Hydrochloride Nanostructured Lipid Nanocarrier Loaded Microneedle Array Patch for Depression, International Journal of Pharmaceutics. (2025) 671, 10.1016/j.ijpharm.2025.125257. 39855281

[164] Pinto M. , Machado C. S. , Barreiro S. et al., Rescuing a Troubled Tolcapone with PEGylated PLGA Nanoparticles: Design, Characterization, and Hepatotoxicity Evaluation, ACS Applied Materials & Interfaces. (2024) 16, no. 17, 21522–21533, 10.1021/acsami.4c00614. 38647198

[165] Machado C. S. , Pinto M. , Aguiar B. et al., Exploring Nanocarriers for Boosting Entacapone Bioavailability: A Journey Through System Characterization and Assessment of Toxicity and Pharmacological and 2D Permeability Paybacks, ACS Applied Materials & Interfaces. (2024) 16, no. 43, 58299–58312, 10.1021/acsami.4c12372. 39432433

[166] Agrawal Y. , Patil K. , Mahajan H. et al., In Vitro and in Vivo Characterization of Entacapone-loaded Nanostructured Lipid Carriers Developed by quality-by-design Approach, Drug Delivery. (2022) 29, no. 1, 1112–1121, 10.1080/10717544.2022.2058651. PMC8986208 35380091

[167] Venkateswarlu G. , Entacapone Nanoemulsion: Formulation Design, Optimization, and Evaluation, 2024.

