# Supplementary material for: Chronic Lead Exposure and Tauopathies: Molecular Mechanisms of Neurotoxicity and Emerging Nanotherapeutic Strategies
Source: J Toxicol. 2026 Oct 13;2026:3248563. doi: 10.1155/jt/3248563 (PMC13660707; doi:10.1155/jt/3248563)
Supplement: Supplementary file 1 [file JT-2026-3248563-s001.docx]

**Supplementary Table S1.** Recent applications of nanoparticle-based systems as delivery carriers for approved drugs used in AD therapy.

| **Drug** | **NCs type** | **NCs composition** | **NCs Size (nm)** | **Route of**  **Administration** | **Main Outcomes** | **Ref.** |
| --- | --- | --- | --- | --- | --- | --- |
| Galantamine | Liposome-embedded scaffold (LEPS) | Functionalized liposomes, chitosan, Eudragit® RSPO, polyvinyl alcohol (PVA) | 100–158 | Not explicitly stated | LEPS enabled sustained galantamine release over 50 days, maintained liposome integrity, preserved intracellular drug activity, showed no significant cytotoxicity, and supported neuronal cell proliferation. | [130] |
|  | Nanospray-dried polymeric nanoparticles | Galantamine hydrobromide, polyacrylic acid, sodium taurodeoxycholate | 185.55 ± 4.3 | Intranasal | Nanoparticles improved mucoadhesion, brain uptake, and anti-inflammatory efficacy in Alzheimer’s mouse model. | [131] |
|  | Chitosan NPs in Poloxamer Hydrogel | Thiolated Chitosan, galantamine - Poloxamer 407 | ~98–100 | Intranasal | Mucoadhesive hydrogel enabled sustained drug release and cellular uptake with high biocompatibility and retention. | [132] |
|  | Liposomes | Soya phosphatidylcholine, cholesterol, and PG | ~100–200 (not specified) | Intranasal | Liposomes were capable of delivering galantamine via the nose-to-brain route with superior pharmacokinetics and AChE inhibition. | [133] |
|  | Polymeric | Galantamine, biodegradable polymer | ~20 (hydrodynamic radius) | Intravenous | Nanoparticles showed high encapsulation (>90%), long-term stability, sustained release, and preserved drug activity with no cytotoxicity. | [134] |
|  | SLNs | Galantamine hydrobromide, biodegradable lipids | <100 | Not specified | SLNs improved galantamine bioavailability 2-fold, enabled >90% sustained release over 24 h, and significantly restored memory in cognitive deficit rats. | [135] |
|  | Chitosan NPs  (TCS-NPs) | Galantamine, thiolated Chitosan | 149.3-150 | Intranasal | TCS-NPs showed superior memory recovery and AChE inhibition in mice vs. oral/nasal solution, supporting nose-to-brain delivery for Alzheimer's therapy. | [136] |
| Donepezil | Polymeric Nanoparticles | PVA, BiFeO₃ magnetic NPs, donepezil hydrochloride | 164 ± 12 | Not specified | Nanoparticles enabled controlled, electrically stimulated release of donepezil with confirmed biocompatibility. | [137] |
|  | PLGA | Donepezil | 40.8 | Not explicitly stated | PLGA nanoparticles enhanced brain uptake of donepezil, showed sustained release following the Hixson–Crowell model, and may improve AD therapy via BBB transport. | [138] |
|  | PLGA | Donepezil hydrochloride | 136.37 | Not explicitly stated | PLGA nanoparticles enhanced drug stability, demonstrated sustained release with Fickian/non-Fickian mechanisms, showed potential BBB penetration, and exhibited significant anti-AChE and anti-BuChE activities, supporting AD therapy development. | [139] |
|  | Chitosan | Donepezil hydrochloride | 180.2 | Intranasal | Chitosan nanoparticles enhanced brain uptake of donepezil via direct nose-to-brain delivery, showed sustained release, and improved AD therapy by bypassing the BBB. | [140] |
|  | Polymeric nanospheres | Donepezil-loaded chitosan (Cs) and chitosan/polyethylene glycol (Cs/PEG) | 136.6 ± 17.5 |  | Polymeric nanospheres provided controlled donepezil release for 12 h, demonstrated cyto safety, and showed potential anti-Alzheimer efficacy in vitro. | [141] |
|  | Chitosan/tripolyphosphate (CS/TPP) nanoparticles embedded in microneedles | Donepezil and Curcumin loaded CS/TPP NPs coated with polysorbate 80, | 42.63 | Transdermal | Nanoparticles enhanced transdermal delivery of donepezil and curcumin with controlled release following Higuchi kinetics, improving drug penetration and efficacy in AD models while ensuring biocompatibility and safety. | [142] |
| Rivastigmine | Polymeric | Rivastigmine and quercetin co-loaded PCL-PEG-PCL nanocarriers surface-modified with valine-conjugated chitosan (VLCS) | 121.83 ± 4.58 | Intraperitoneal | PCL-PEG-PCL nanocarriers co-loaded with rivastigmine and quercetin enhanced memory restoration, improved GSH levels, modulated AD-related gene expression, and may improve AD therapy via LAT-1-mediated BBB transport. | [143] |
|  | Liposomes and PLGA | Rivastigmine-loaded liposomes and PLGA nanoparticles | NA | Intranasal | Liposomal rivastigmine enhanced brain delivery with rapid onset, higher bioavailability, prolonged half-life, and significantly improved memory performance in AD models via nasal route. | [144] |
|  | Nanostructured Lipid Carriers (NLCs) | Rivastigmine-loaded NLCs incorporated in in situ nasal gel | 123.2 ± 2.3 | Intranasal and Intravenous | NLCs enhanced brain uptake of rivastigmine via nose-to-brain delivery, showed sustained release, improved memory recovery, and demonstrated safety for nasal application. | [145] |
|  | Chitosan Nanoparticles | Rivastigmine-loaded chitosan-tripolyphosphate NPs with Tween 80 | 291.6 ± 7.70 to 490.6 ± 7.42 | Transdermal (via Nano-gel) | Spherical NPs with high EE (89.80%) and drug loading (40.81%); improved skin permeation and sustained release; non-irritant and stable; suitable for brain targeting. | [146] |
|  | mesoporous silica nanoparticles | Calcium-doped mesoporous silica nanoparticles functionalized with polysorbate-80 (PS), loaded with rivastigmine (RV | NA | Intravenous | PS-functionalized MSNs increased brain-to-plasma ratio, brain uptake clearance, and plasma half-life of RV; confirmed BBB penetration and enhanced brain delivery. | [147] |
|  | Polymeric NC  (simulated) | Poly(n-butyl cyanoacrylate) nanoparticles encapsulating rivastigmine | NA (MD simulation-based) | NA | Molecular dynamics simulations revealed that drug loading plateaus due to limited encapsulation; excess drug accumulates on surface. Encapsulation depends on polymer–drug interactions and polymer structure. | [148] |
|  | Lipid-based NCs in gel | Rivastigmine-loaded nanostructured lipid carriers (NLC) and nanoemulsions in thermosensitive in situ nasal gels | NLC: 114.00 ± 1.91 NE: 135.80 ± 0.50 | Intranasal | High encapsulation efficiency (NLC: 95.13%, NE: 89.86%), low cytotoxicity, improved nasal mucoadhesion, 2× higher olfactory drug deposition (8% vs 4%), spherical shape, and long-term stability. Promising alternative for enhanced AD therapy. | [149] |
|  | Mucoadhesive Microspheres | Rivastigmine-loaded ethylcellulose–chitosan microspheres, surface-functionalized with lectin | ~19.9 µm | Intranasal | Entrapment efficiency: 77.8%; Lectin-functionalized microspheres showed significantly higher bioadhesion (64% vs 29%), improved memory retention in rats. | [150] |
| Memantine | Chitosan | Memantine hydrochloride-loaded chitosan nanocrystals (via ionic gelation with STPP) | 152.63 ± 12.95 nm | NA (highly intranasal) | High drug loading (DL: 98.44 ± 3.31%) and entrapment efficiency (EE: 78.7 ± 3.11%). Sustained release up to 24 h with initial burst (86.5%). No cytotoxicity or nasal tissue damage. Promising for in vivo AD therapy. | [151] |
|  | PEG-coated PLGA nanostructure | PEG-coated poly D,L-lactic-co-glycolic acid | ~150 | NA | Sustained drug release (Korsmeyer–Peppas model), effective BBB penetration, enzymatic inhibition (AChE, BuChE, β-secretase), improved cognition in animal model | [152] |
|  | Nanoscaffolds | PLGA, Pluronics F-127, Memantine | 100-200 | NA | PLGA nanoscaffolds enhanced memantine loading and porosity, showed controlled release, inhibited enzymes and inflammatory cytokines, and may improve AD therapy. | [153] |
|  | Polymeric NPs | Memantine | 58.04 | Intranasal | Polymeric nanoparticles enhanced brain uptake of memantine via the intranasal route, showed sustained 24-hour release, and may improve AD therapy with minimal cytotoxicity. | [154] |

**Supplementary Table S2.** Recent applications of nanoparticle-based systems as delivery carriers for approved drugs used in PD therapy.

| **Drug** | **NCs type** | **NCs composition** | **NCs Size (nm)** | **Route of**  **Administration** | **Main Outcomes** | **Ref.** |
| --- | --- | --- | --- | --- | --- | --- |
| Levodopa | Gold nanorods | Gold nanorods (AuNRs) coated with a ROS-responsive levodopa prodrug (PCB-Se-Se-LD) and functionalized with angiopep-2 (ANG) | 25 × 113 | Intravenous | Au-PCBLD-ANG nanocarriers enabled brain-targeted levodopa delivery via photothermal and ANG-mediated BBB crossing, enhancing accumulation, reducing ROS, and providing neuroprotection | [155] |
|  | Gold NPs | AuNPs functionalized with PEG, 1-adamantylamine (Ad), 1-adamantylglycine (AdGly), and peptidoglycan monomer (PGM) | 50.3 -111.3 | NA | PGM-functionalized AuNPs were capable of binding and loading L-DOPA and dopamine efficiently, even in the presence of serum proteins, showing potential as nanocarriers for Parkinson’s disease therapy. | [156] |
|  | Gold NPs | Functionalized with glutathione (GSH), polyethylene glycol (PEG), bovine serum albumin (BSA); and loaded with levodopa | 10-20 | NA | Functionalized AuNPs were capable of binding and stabilizing catechol drugs and showed ability to cross the in vitro BBB model, indicating potential for brain-targeted delivery in Parkinson’s therapy. | [157] |
|  | Chitosan | Levodopa and selegiline loaded chitosan NPs | 62.18 | NA | Chitosan nanoparticles could deliver both levodopa and selegiline simultaneously with efficient entrapment and rapid in vitro release, indicating potential for Parkinson’s disease treatment. | [158] |
|  | Silica NPs | Lactoferrin-modified lipid-coated mesoporous silica nanoparticles (Lf-lip@LC-MSNs) loaded with levodopa and curcumin | 102.67 | intraperitoneally | Lf-lip@LC-MSNs were capable of co-delivering levodopa and curcumin with enhanced brain targeting, reduced oxidative stress and α-synuclein aggregation, and improved motor recovery in PD models. | [159] |
|  | Selenium nanoparticles (SeNPs) | Coated with polysaccharides (chitosan, dextran), functionalized with catechol drugs (levodopa, dopamine) | 50–120 | NA | Functionalized SeNPs were capable of binding catechol drugs and permeating the in vitro BBB model, offering biocompatible and effective delivery systems for Parkinson’s disease. | [157] |
| MAOB inhibitors | Chitosan | Selegiline hydrochloride-loaded chitosan nanoparticles stabilized with Tween | 63.1 | Intranasal | Chitosan nanoparticles were capable of delivering selegiline via the nose-to-brain route, improving bioavailability, reducing oxidative stress, and reversing PD symptoms with prolonged release. | [160] |
|  | Nanoemulsion | Selegiline | 61.43±4.10 | Intranasal | Selegiline nanoemulsion was capable of nose-to-brain delivery, enhancing brain dopamine levels, antioxidant enzyme activity, and bioavailability while reducing oxidative stress in Parkinson’s models. | [161] |
|  | Liposomes | Co-loaded with selegiline and α-synuclein-targeting siRNA (siSNCA2); coated with human serum albumin (HSA) | 136.5 ± 10.3 | Intranasal | Albumin-coated liposomes were capable of delivering selegiline and siRNA via the nose-to-brain route, improving motor function, reducing MAO-B and α-synuclein, and enhancing brain targeting in PD models. | [162] |
|  | Nanostructured lipid carriers | Selegiline-loaded NLCs incorporated into microneedle array patch | 158.71 ± 0.56 | Transdermal | Nanostructured lipid carriers were capable of transdermal delivery of selegiline with sustained release, prolonged half-life, enhanced brain bioavailability, and no observed toxicity, improving therapeutic efficacy. | [163] |
| COMT | PLGA NPs | PEGylated PLGA nanoparticles loaded with tolcapone | <200 | NA | PLGA nanoparticles were capable of delivering tolcapone with reduced cytotoxicity, maintained COMT inhibition, and protection against ATP depletion and ROS, offering a safer alternative to free tolcapone. | [164] |
|  | PLGA NPs | PEGylated PLGA nanoparticles loaded with entacapone | <200 | NA | PLGA nanoparticles were capable of delivering entacapone with improved permeability and stability, maintaining COMT inhibition and reducing cytotoxicity. | [165] |
|  | Nanostructured lipid carriers | PEGylated nanostructured lipid carriers loaded with entacapone | <200 | NA | NLCs were capable of delivering entacapone with improved permeability and maintained COMT inhibition, though with observed cytotoxicity in HepG2 cells | [165] |
|  | Nanostructured lipid carriers (NLCs) | Entacapone-loaded NLCs developed via Quality by Design | NA | Oral | LCs were capable of delivering entacapone orally with improved half-life, bioavailability, and prolonged therapeutic action, addressing its poor dissolution and first-pass metabolism limitations | [166] |
|  | Nanoemulsion | Entacapone | 120.8 ± 1.9 | Oral | Nanoemulsions were capable of delivering entacapone orally with enhanced solubility, uniform particle size, and improved release profile, offering a stable formulation for Parkinson’s therapy. | [167] |
